# Synthesis, characterization and *in vitro* antitrypanosomal activities of new carboxamides bearing quinoline moiety

**DOI:** 10.1371/journal.pone.0191234

**Published:** 2018-01-11

**Authors:** David Izuchukwu Ugwu, Uchechukwu Chris Okoro, Narendra Kumar Mishra

**Affiliations:** 1 Medicinal Chemistry Unit, Department of Pure and Industrial Chemistry, University of Nigeria, Nsukka, Nigeria; 2 Department of Chemistry, Indian Institute of Technology, Kanpur, India; Istituto di Genetica Molecolare, ITALY

## Abstract

The reported toxicities of current antitrypanosomal drugs and the emergence of drug resistant trypanosomes underscore the need for the development of new antitrypanosomal agents. We report herein the synthesis and antitrypanosomal activity of 24 new amide derivatives of 3-aminoquinoline, bearing substituted benzenesulphonamide. Nine of the new derivatives showed comparable antitrypanosomal activities at IC_50_ range of 1–6 nM (melarsoprol 5 nM). Compound **11n** and **11v** are more promising antitrypanosomal agents with IC_50_ 1.0 nM than the rest of the reported derivatives. The novel compounds showed satisfactory predicted physico-chemical properties including oral bioavailability, permeability and transport properties.

## Introduction

Trypanosomes are parasitic protozoa that cause Chagas disease in central and South America and sleeping sickness in sub-Saharan Africa leading to morbidity and mortality of millions of people [1]. *Trypanosoma brucei gambiense* and *T*. *b*. *rhodesiense* are the etiological agents of sleeping sickness in humans in sub-Saharan Africa. About half a million people are infected with sleeping sickness leading to the death of almost 100,000 people annually [2]. Despite the fact that in the past 24 years, the number of incidence for both Chagas and human African trypanosomiasis (HAT) has significantly declined, primarily due to vector control initiatives [3], the development of new chemotherapeutic agent for the treatment of sleeping sickness and Chagas disease is urgently needed because of the undesirable toxic side effects of known antitrypanosomal drugs, and the emergence of drug-resistant trypanosomes.

Benznidazole (**1**), melarsoprol (**2**), pentamidine (**3**) and nifurtimox (**4**) are the leading chemotherapeutic agent used in the treatment of Chagas and sleeping sickness. Benznidazole (**1**) is primarily used for the treatment of Chagas disease. Melarsoprol (**2**) has a high number of associated side effects [4] including but not limited to brain dysfunction, convulsion, numbness, rashes, kidney and liver problems [5]. About 1–5% of people die from adverse effects related to melarsoprol treatment [6]. Pentamidine (**3**) is usually applied at the early stage of the parasite before it crosses to central nervous system. It is not recommended in early pregnancy because of the associated side effects [7]. The most common side effects of concern is wheezing, hypotension, severe arrhythmias and heart failure [8, 9]. Pentamidine has also been linked to azotemia, renal failure, hepatomegaly, hepatitis, seizures and hypoglycemia [9]. In addition to the side effects of pentamidine, it is also costly with the inhalation powder costing up to 122.84 USD and a vial for injection costing 45.31 USD in the United States [10]. Nifurtimox (**4**) takes 30 to 60 days administration to treat Chagas disease [11]. This long term use does increase chances of adverse effects like gastrointestinal and neurological disorder [12]. It is also reported that administration of nifurtimox in sleeping sickness leads to relapsing in half of the patients treated [13]. The use of nifurtimox in pregnancy is usually avoided because of the associated side effects [14]. Nifurtimox has also been linked to central nervous system disturbances, peripheral neuropathy, confusion, seizures, impotency, depression and numbness [15–17]. Eflornithine (**5**) is the only new drug introduced for the treatment of sleeping sickness but was ineffective against *T*. *b*. *rhodesiense* [18].

As there is no immediate prospect for vaccines, chemotherapy is the only way to fight trypanosomes. This enzyme is responsible for the major proteolytic activity of all life cycle stages of this parasite [19]. Brucipain is localized in the lysosomes of blood stream forms of *T*. *brucei* [20]. It induces calcium activation signals that allows blood brain barrier to open up to parasite crossing [21]. This makes this enzyme a good target for the treatment of human African trypanosomiasis.

In line with the call for the synthesis of new chemotherapeutic agent for the treatment of human African trypanosomiasis, Ajibade and Kolawole [22] reported antitrypanosomal activities of some sulfadiazine complex. Papadopoulou et al [23] also reported the antitrypanosomal activities of some amides and sulphonamides. Some of the derivatives reported by this group were more potent up to 58-fold than benznidazole (**1**).

**Fig 1:**
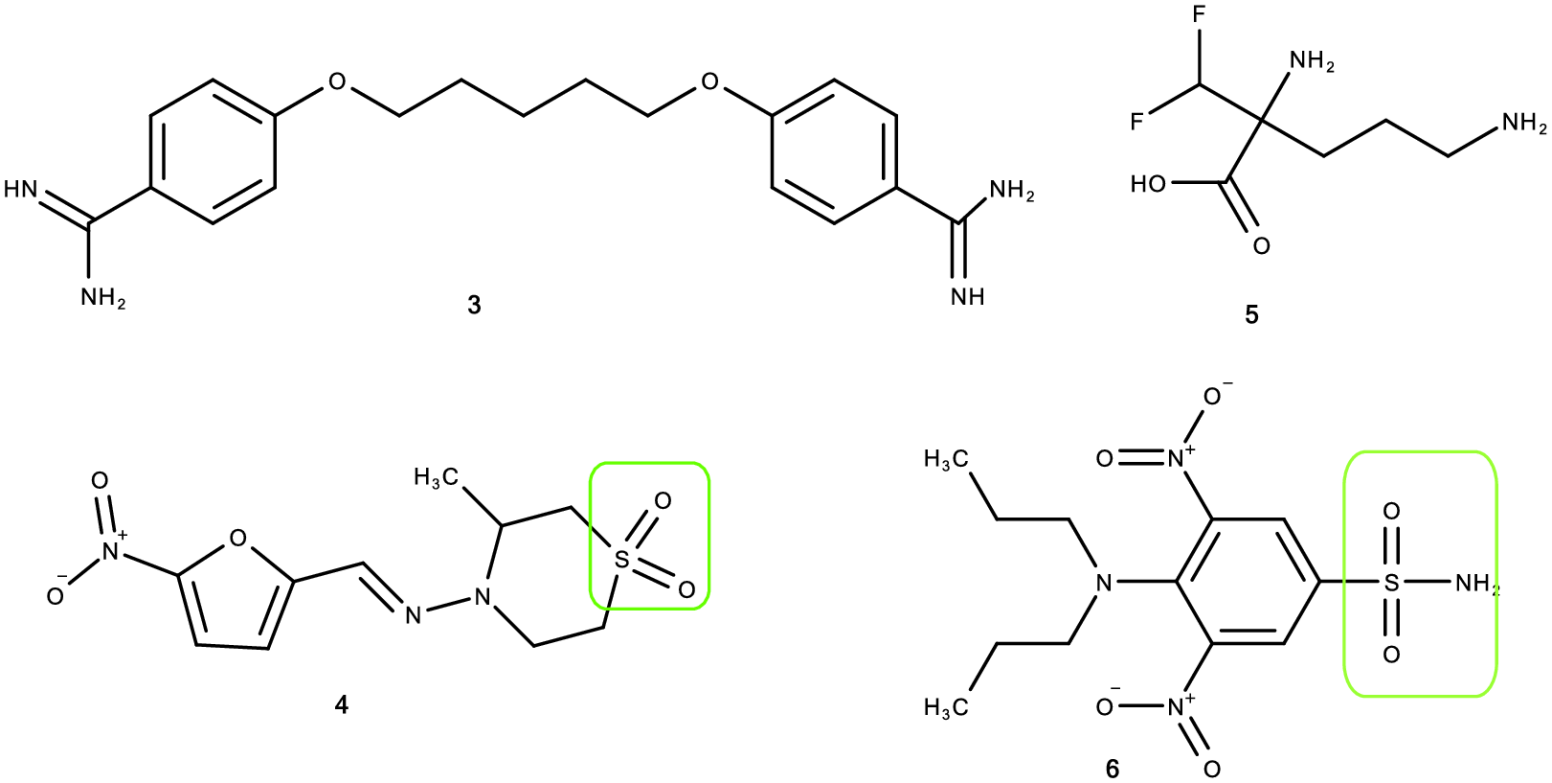
Examples of commercial antitrypanosomal agents. A representation of some compounds used as antitrypanosomal agent.

Since benznidazole (**1**) contains an amide functionality and nifurtimox (**4**) contains a sulfone group, synergistic antitrypanosomal activity arising from successful combination of amide of benznidazole and sulfone of nifurtimox motivated this research. This expectation was supported by the work of Papadopoulou which showed comparable antitrypanosomal activity when amide groups were replaced with sulphonamides. This finding suggests that successful combination of the functionalities would possibly lead to a synergy. Werbovetz et al [24] reported oryzalin (**6**) as the most active antiparasitic agent. It showed GI_50_ values of 0.41 and 0.73 μM *in vitro* against two strains of *T*. *brucei* with a selectivity index of 40–80 thereby making it a promising lead. This compound possess both amide and sulphonamide functionality. The structures of most widely used commercial antitrypanosomal drugs are shown in Fig 1.

From the forgoing, there is need to synthesize new antitrypanosomal agents that will retain high potency of melarsoprol and other commercial antitrypanosomal agents while having an improved toxicity profile.

As a continuation of our search for lead antitrypanosomal agents, we report in this work fascinating antitrypanosomal activities of newly synthesized carboxamides from unactivated carboxylic acids bearing sulphonamide functionality. The synergy arising from the successful incorporation of carboxamide group in compounds containing sulphonamide pharmacophore is exploited in this research.

## Experimental

### Instrumentation

All reactions requiring inert atmosphere were carried out under nitrogen atmosphere. Drying of solvents was achieved using molecular sieve for 48 h. All reagents were purchased from commercial suppliers, Aldrich, Merck, Fluka, Avra, SD fine and Alfa Aesar. Thin layer chromatography was carried out using silica plates purchased from Avra. The plates were visualized under UV light (popular India). FT-IR spectroscopy of the compounds were run in PerkinElmer Spectrum version 10.03.06 and the bands presented in wavenumber. Proton and carbon-13 NMR spectroscopy were run in DMSOd_6_ and CD_3_OD, unless otherwise stated on either Jeol 500 MHz or 400 MHz. The chemical shifts were reported in part per million with reference to tetramethylsilane. Mass spectroscopy were carried out using micro TOF electrospray time of flight (ESI-TOF) mass spectrometer (Aerodyne Research Inc. USA), sodium formate was used as the calibrant. All experiments were carried out at Prof. Sandeep Verma’s Laboratory, department of Chemistry, Indian Institute of Technology, Kanpur. Melting points were determined using digital melting point apparatus (Stuart, SMP20) and were uncorrected.

### General procedure for the synthesis of substituted benzenesulphonamoyl alkanamides (7a-x)

Sodium carbonate (Na_2_CO_3_, 1.590 g, 15 mmol) was added to a solution of amino acids (**6a-h**, 12.5 mmol) in water (15 mL) with continuous stirring until all the solutes dissolved. The solution was cooled to -5°C and an appropriate benzenesulphonyl chloride (**5a-c**, 15 mmol) was added in four portions over a period of 1 h. The slurry was further stirred at room temperature for 4 h. The progress of the reaction was monitored by using TLC (MeOH/DCM, 1:9). Upon completion, the mixture was acidified using 20% aqueous hydrochloric acid to pH 2. The crystals was filtered via suction and washed with pH 2.2 buffer. The pure products **(7a-x)** were dried over self-indicating fused silica gel in a desiccator.

#### 2-(4-methylphenylsulphonamido) acetic acid (7a)

The amino acid was glycine, yield (2.8410 g, 99.34%), appearance white needles, mp, 88.4–88.6°C, FTIR (KBr, cm^-1^): 3448 (OH of COOH), 3277 (NH), 2957 (C-H aliphatic), 1730 (C = O), 1598, 1440 (C = C), 1354, 1321 (2S = O), 1185 (SO_2_-NH), 1111, 1094 (C-N, C-O). ^1^H NMR (500 MHz, DMSO-d_6_) δ: 7.89 (t, J = 6.3 Hz, 1H, NH), 7.64 (d, J = 8.6 Hz, 2H, ArH), 7.33 (d, J = 8.05 Hz, 2H, ArH), 3.51 (d, J = 5.7 Hz, CH_2_), 2.34 (s, 3H, CH_3_). ^13^C NMR (500 MHz, DMSO-d_6_) δ: 170.8 (C = O), 143.1, 138.4, 130.0, 127.1 (aromatic carbons), 44.3 (CH_2_), 21.5 (CH_3_). HRMS-ESI (m/z): 228.0410 (M-H)^-^, calculated, 228.0408.

#### 2-(4-methylphenylsulphonamido)-3-phenylpropanoic acid (7b)

The amino acid was L-phenylalanine, yield (3.9848 g, 99.93%), appearance, off-white powder, mp, 135.10–135.40°C FTIR (KBr, cm^-1^): 3439 (OH of COOH), 3322 (NH), 3025 (C-H aromatic), 2926 (C-H aliphatic), 1713 (C = O), 1597, 1496, 1456 (C = C), 1344, 1331 (2S = O), 1189, 1157 (SO_2_NH), 1136, 1090, 1052 (C-N, C-O). ^1^H NMR (DMSO-d6, 500 MHz) δ: 8.12 (d, J = 9.15 Hz, 1H, NH), 7.46 (d, J = 7.45 Hz, 2H, ArH), 7.42 (d, J = 8.6 Hz, 2H, ArH), 7.14 (m, 5H, ArH), 3.82 (dd, J = 3.45, 2.85 Hz, 1H, CH), 2.89 (dd, J = 5.75, 5.75 Hz, 1H, CH_a_ of CH_2_), 2.67 (dd, J = 8.6, 8.6 Hz, 1H, CH_b_ of CH_2_), 2.31 (m, 3H, CH_3_). ^13^C NMR (DMSO-d6, 500 MHz) δ: 172.8 (C = O), 142.8, 138.7, 137.3, 129.7, 128.7, 126.9, 126.8, 126.0 (eight aromatic carbons), 57.9, 38.0, 21.5 (three aliphatic carbons). HRMS-ESI (m/z): 320.0958 (M+H)^+^, calculated, 320.0951.

#### 3-(1H-indol-2-yl)-2-{[(4-methylphenyl)sulphonyl]amino}propanoic acid (7c)

The amino acid was L-tryptophan, yield (4.3814 g, 97.880%), appearance, yellowish liquid, FTIR (KBr, cm^-1^): 3385, 3302 (2NH), 2921, 2858 (C-H aliphatic), 1750 (C = O), 1618, 1598, 1494, 1457, 1429 (C = C), 1321, 1291 (2S = O), 1163, 1131 (SO_2_NH), 1082, 1019 (C-N, C-O). ^1^H NMR (DMSO-d_6_, 500 MHz) δ: 10.73 (s, 1H, NH of indole), 8.07 (d, J = 8.60 Hz, 1H, NH of SO_2_NH), 7.67 (d, J = 8.05 Hz, 1H, ArH), 7.42 (d, J = 8.60 Hz, 2H, ArH), 7.33 (d, J = 8.00 Hz, 1H, ArH), 7.25 (t, J = 8.05 Hz, 1H, ArH), 7.13 (d, J = 8.00 Hz, 2H, ArH), 7.05–7.01 (m, 2H, ArH), 6.90 (m, 1H, ArH), 3.84 (dd, J = 7.45, 8.00 Hz, 1H, CH), 3.00 (dd, J = 6.3, 6.90 Hz, 1H, CH_a_, CH_2_), 2.81 (dd, J = 7.45, 7.45 Hz, 1H, CH_b_ of CH_2_), 2.28 (s, 3H, CH_3_-Ar). ^13^C NMR (DMSO-d_6_, 500 MHz) δ: 173.2 (C = O), 142.7, 138.5, 136.6, 129.6, 127.4, 126.8, 124.5, 121.3, 118.8, 118.3, 111.9, 109.4 (aromatic carbons), 66.9, 57.1, 21.5 (aliphatic carbons). HRMS-ESI (m/z): 359.1060 (m+H)^+^, calculated, 359.1064.

#### 4-Methyl-2-{[(4-methylphenyl)sulfonyl]amino}pentanoic acid (7d)

The amino acid was L-leucine, yield (3.502 g, 98.26%), appearance, off-white powder, mp, 114.10–114.50°C. FTIR (KBr, cm^-1^): 3423 (OH of CO_2_H), 3279 (NH), 2949 (C-H aromatic), 2872 (C-H aliphatic), 1706 (C = O), 1598, 1497, 1458, 1420 (C = C), 1384, 1339 (2S = O), 1168, 1152 (SO_2_NH), 1122, 1091, 1020 (C-N, C-O). ^1^H NMR (DMSO-d_6_, 500 MHz) δ: 7.97 (d, J = 8.60 Hz, 1H, NH), 7.61 (d, J = 8.55 Hz, 2H, Ar-H), 7.31 (d, J = 8.55 Hz, 2H, ArH), 3.60 (m, 1H, CH-CO_2_H), 2.33 (s, 3H, CH_3_-Ar), 1.53 (m, 1H, CH), 1.34 (m, 2H, CH_2_), 0.76 (d, J = 6.85 Hz, 3H, CH_3_), 0.65 (d, J = 6.30 Hz, 3H, CH_3_). ^13^C NMR (DMSO-d_6_, 500 MHz) δ: 173.8 (C = O), 142.9, 138.9, 129.9, 128.6, 127.0, 126.0 (aromatic carbons), 54.5, 41.5, 24.4, 23.1, 21.6 (aliphatic carbons). HRMS-ESI (m/z): 286.1986 (M+H)^+^, calculated, 286.1988.

#### 3-methyl-2-{[(4-methylphenyl)sulfonyl]amino}pentanoic acid (7e)

The amino acid was L-isoleucine, yield (3.412 g, 95.74%), appearance, off-white, mp, 130.10–130.50°C, FTIR (KBr, cm^-1^): 3280 (NH), 2970, 2934 (C-H Ar-H), 2883 (C-H aliphatic), 1710 (C = O), 1599, 1496, 1460 (C = C), 1385, 1334 (2S = O), 1185, 1161 (SO_2_NH), 1092, 1058, 1020 (C-N, C-O). ^1^H NMR (DMSO-d_6_) δ: 7.87 (d, J = 9.2 Hz, 1H, NH), 7.61 (d, J = 8.05 Hz, 2H, ArH), 7.31 (d, J = 8.05 Hz, 2H, ArH), 3.49 (dd, J = 6.30, 6.30 Hz, 1H, CH-CO_2_H), 2.33 (s, 3H, CH_3_-Ar), 1.60 (m, 1H, CH), 1.31 (m, 1H, CH_a_ of CH_2_), 1.05 (m, 1H, CH_b_ of CH_2_), 0.73 (dt, J = 6.85, 7.45H, 6H, 2CH_3_). ^13^C NMR (DMSO-d_6_, 500 MHz) δ: 172.7 (C = O), 142.9, 138.9, 129.8, 127.1 (aromatic carbons), 60.5, 37.5, 24.9, 21.5, 15.9, 11.4 (aliphatic carbons). HRMS-ESI (m/z): 284.0765 (m-H)^-^, calculated, 284.0769.

#### 3-Methyl-2-(4-methylphenylsulphonamido)butanoic acid (7f)

The amino acid was L-valine, yield (3.2849 g, 95.56%), appearance, off-white, mp, 121.9–122.20°C, FTIR (KBr, cm^-1^): 3293 (NH), 2970 (C-H aliphatic), 1708 (C = O), 1598, 1466, 1419 (C = C), 1333, 1289 (2S = O), 1161 (SO_2_NH), 1089 (C-N or C-O). ^1^H NMR (DMSO-d_6_, 500 MHz) δ: 7.86 (d, J = 9.75 Hz, 1H, NH), 7.65 (m, 2H, ArH), 7.28 (m, 2H, ArH), 3.45 (dd, J = 5.70, 5.75Hz, 1H, CH-CO_2_H), 2.31 (m, 3H, CH_3_-Ar), 1.88 (m, 1H, CH), 0.71 (m, 6H, 2CH_3_). ^13^C NMR (DMSO-d_6_, 500 MHz) δ: 172.7 (C = O), 142.9, 138.9, 129.8, 127.1, 126.2 (aromatic carbons), 61.7, 30.9, 21.5, 19.5, 18.4 (aliphatic carbons). HRMS-ESI (m/z): 271.0881 (M^+^), calculated, 271.0889.

#### 4-Hydroxy-1-tosylpyrrolidine-2-carboxylic acid (7g)

The amino acid was L-hydroxyproline, yield (3.564 g, 99.86%), appearance, white needles, mp, 98.40–98.60°C. FTIR (KBr, cm^-1^): 3524 (OH), 2931 (C-H aliphatic), 1708 (C = O), 1600, 1444 (C = C), 1347, 1332 (2S = O), 1200, 1158 (SO_2_N), 1090, 1075 (C-N or C-O). ^1^H NMR (DMSO-d_6_, 400 MHz) δ: 7.65 (d, J = 8.00 Hz, 2H, ArH), 7.37 (d, J = 8.05 Hz, 2H, ArH), 4.18 (s, 1H, O-H), 4.01 (t, J = 8.05 Hz, 1H, CH-CO_2_H), 3.42 (m, 1H, CH-OH), 3.05 (d, J = 10.3 Hz, 2H, CH_2_), 2.36 (s, 3H, CH_3_), 1.91 (t, 4.6 Hz, 2H, CH_2_). ^13^C NMR (DMSO-d_6_, 400 MHz) δ: 173.8 (C = O), 143.7, 135.0, 130.1, 127.9 (aromatic carbons), 68.9, 60.2, 56.8, 21.5 (aliphatic carbons). HRMS-ESI (m/z): 286.1097 (m+H)^+^, calculated, 286.1097.

#### 1-Tosylpyrrolidine-2-carboxylic acid (7h)

The amino acid was L-proline, yield (3.2989 g, 98.09%), appearance, yellowish powder, mp, 50.4–50.80°C, FTIR (KBr, cm^-1^): 3415 (OH of COOH), 2957 (C-H aliphatic), 1737 (C = O), 1619, 1597, 1494, 1449 (C = C), 1345, 1306 (2S = O), 1199 (SO_2_N), 1159, 1095, 1013 (C-N, C-O). ^1^H NMR (DMSO-d_6_, 500 MHz) δ: 7.65 (d, J = 8.6 Hz, 2H, ArH), 7.35 (d, J = 8.00 Hz, 2H, ArH), 4.04 (dd, J = 4.55, 5.15 Hz, 1H, CH-COOH), 3.29 (dd, J = 9.75, 5.15Hz, 1H, CH_a_ of CH_2_-N), 3.09 (dd, J = 6.85, 7.45 Hz, 1H, CH_b_ of CH_2_-N), 2.31 (s, 3H, CH_3_-Ar), 1.77 (m, 3H), 1.48 (t, J = 5.15 Hz, 1H). ^13^C NMR (DMSO-d_6_, 500 MHz) δ: 173.7 (C = O), 143.9, 135.1, 130.3, 127.6 (aromatic carbons), 66.9, 48.9, 30.9, 24.7, 21.4 (aliphatic carbons). HRMS-ESI (m/z): 269.0726 (m+H)^+^, calculated, 269.0726.

#### 2-Benzenesulphonamido acetic acid (7i)

The amino acid was glycine, yield (2.6856 g, 99.61%), appearance, off-white powder, mp, 170.80–170.90°C. FTIR (KBr, cm^-1^): 3317 (NH), 3060, 2974 (C-H aromatic), 2946 (C-H aliphatic), 1728 (C = O), 1587, 1451, 1428, 1412 (C = C), 1318, 1247 (S = O), 1158, 1130 (SO_2_NH), 1095, 1077, 1012 (C-N, C-O). ^1^H NMR (DMSO-d_6_, 500 MHz) δ: 8.01 (t, J = 12.00 Hz, 1H, NH), 7.76 (d, J = 7.45 Hz, 2H, ArH), 7.59 (t, J = 7.45 Hz, 1H, ArH), 7.54 (t, J = 8.00 Hz, 2H, ArH), 3.55 (d, J = 6.30 Hz, 2H, CH_2_). ^13^C NMR (DMSO-d_6_, 500 MHz) δ: 170.7 (C = O), 141.2, 132.9, 129.6, 126.9 (aromatic carbons), 44.5 (aliphatic carbon). HRMS-ESI (m/z): 216.1252, calculated 216.1257.

#### 2-Benzenesulphonamido-3-phenylpropanoic acid (7j)

The amino acid was L-phenylalanine, yield (3.8169 g, 100%), appearance, off-white powder, mp, 129.10–129.50°C. FTIR (KBr, cm^-1^): 3342 (NH), 3195, 3029 (C-H aromatic), 2968 (C-H aliphatic), 1735 (C = O), 1697, 1496, 1447 (C = C), 1375, 1347 (2S = O), 1170, 1108 (SO_2_NH), 1093, 1028 (C-N, C-O). ^1^H NMR (DMSO-d_6_, 500 MHz) δ: 8.25 (d, J = 8.85 Hz, 1H, NH), 7.52 (m, 3H, ArH), 7.40 (t, J = 7.45 Hz, 2H, ArH), 7.15 (m, 3H, ArH), 7.08 (d, J = 7.15 Hz, 2H, ArH), 3.84 (ddd, J = 6.00, 5.75, 6.30 Hz, 1H, CH-CO_2_H), 2.90 (dd, J = 5.75, 5.75 Hz, 1H, CH_a_ of CH_2_), 2.67 (dd, J = 9.00, 9.00 Hz, 1H, CH_b_ of CH_2_). ^13^C NMR (DMSO-d_6_, 500 MHz) δ: 172.8 (C = O), 141.6, 137.3, 132.6, 129.7, 129.4, 128.7, 127.1, 126.7 (aromatic carbons), 57.9, 38.3 (aliphatic carbons). HRMS-ESI (m/z): 304.0624 (M-H)^-^, calculated 304.0628.

#### 2-Benzenesulphonamido-3-(1H-indol-3-yl)propanoic acid (7k)

The amino acid was L-tryptophan, yield (4.2805 g, 99.43%), appearance, faint yellow powder, mp, 106.40–106.70°C. FTIR (KBr, cm^-1^): 3366, 3311 (2NH), 3061 (C-H aromatic), 2936 (C-H aliphatic), 1746 (C = O), 1619, 1550, 1451, 1430 (C = C), 1323, 1235 (2S = O), 1214, 1160 (SO_2_NH), 1127, 1091, 1012 (C-N, C-O). ^1^H NMR (DMSO-d_6_, 500 MHz) δ: 10.78 (s, 1H, NH of indole), 8.22 (d, J = 8.60 Hz, 1H, NH), 7.57 (m, 2H, ArH), 7.48 (t, J = 6.85 Hz, 1H, ArH), 7.37 (m, 2H, ArH), 7.27 (d, J = 8.60 Hz, 2H, ArH), 7.01 (m, 2H, ArH), 6.90 (t, J = 7.40 Hz, 1H, ArH), 3.89 (m, 1H, ArH), 3.03 (dd, J = 6.85, 6.85 Hz, 1H, CH_a_ of CH_2_), 2.83 (dd, J = 8.05, 7.45 Hz, 1H, CH_b_ of CH_2_). ^13^C NMR (DMSO-d_6_, 500 MHz) δ: 173.1 (C = O), 141.5, 136.6, 132.6, 129.2, 127.5, 126.7, 124.5, 121.4, 118.9, 118.4, 111.9, 109.4 (aromatic carbons), 57.2, 28.8 (aliphatic carbons). HRMS-ESI (m/z): 344.0835 (M^+^), calculated 344.0831.

#### 2-Benzenesulphonamido-4-methylpentanoic acid (7l)

The amino acid was L-leucine, yield (3.0592 g, 90.20%), appearance, off-white powder, mp, 105.60°C. FTIR (KBr, cm^-1^): 3248 (NH), 3068 (C-H aromatic), 2959, 2921, 2874 (C-H aliphatic), 1712 (C = O), 1632, 1469, 1450, 1428 (C = C), 1327, 1314 (2S = O), 1184, 11670 (SO_2_NH), 1092, 1077 (C-N, C-O). ^1^H NMR (DMSO-d_6_, 400MHz) δ: 8.09 (d, J = 8.72 Hz, 1H, NH), 7.72 (t, J = 6.88 Hz, 2H, ArH), 7.54 (m, 3H, ArH), 3.60 (m, 1H, CH-CO_2_H), 1.50 (m, 1H, CH), 1.33 (m, 2H, CH_2_), 0.70 (m, 6H, CH_3_). ^13^C NMR (DMSO-d_6_, 400MHz) δ: 173.8 (C = O), 141.6, 132.8, 129.5, 126.9, 126.1 (aromatic carbons), 54.5, 41.4, 24.4, 23.1, 21.5 (aliphatic carbons). HRMS-ESI (m/z): 272.1982 (M+H), calculated, 272.1983.

#### 2-Benzenesulphonamido-3-methylpentanoic acid (7m)

The amino acid was L-isoleucine, yield (3.1052 g, 91.55%), appearance, off-white powder, mp, 148.00–148.30°C. FTIR (KBr, cm^-1^): 3295 (NH), 2968 (C-H aromatic), 2936, 2883 (C-H aliphatic), 1699 (C = O), 1585, 1450, 1416 (C = C), 1340, 1384 (2S = O), 1168 (SO_2_NH), 1092, 1026 (C-N, C-O). ^1^H NMR (DMSO-d_6_, 400 MHz) δ: 8.00 (d, J = 9.16 Hz, 1H, NH), 7.72 (d, J = 8.60 Hz, 2H, ArH), 7.54 (m, 3H, ArH), 3.50 (dd, J = 7.45, 7.45 Hz, 1H, CH-CO_2_H), 1.60 (m, 1H, CH), 1.30 (m, 1H, CH_a_ of CH_2_), 1.04 (m, 1H, CH_b_ of CH_2_), 0.71 (m, 6H, 2CH_3_). ^13^C NMR (DMSO-d_6_, 400MHz) δ: 172.7 (C = O), 141.6, 132.8, 129.4, 127.0 (aromatic carbons), 60.5, 37.4, 24.9, 15.9, 11.4 (aliphatic carbons). HRMS-ESI (m/z): 271.0879 (M^+^), calculated, 271.0878.

#### 2-Benzenesulphonamido-3-methylbutanoic acid (7n)

The amino acid was L-valine, yield (3.2030 g, 99.68%), appearance, white needles, mp, 143.60–143.90°C. FTIR (KBr, cm^-1^): 3418 (OH CO_2_H), 3302 (NH), 2969 (C-H aliphatic), 1703 (C = O), 1585, 1451 (C = C), 1338, 1294 (2S = O), 1169, 1143 (SO_2_NH), 1093, 1042 (C-N, C-O). ^1^H NMR (DMSO-d_6_, 400MHz) δ: 7.97 (d, J = 9.16 Hz, 1H, NH), 7.73 (d, J = 6.88 Hz, 2H, ArH), 7.53 (m, 3H, ArH), 3.46 (dd, J = 6.44, 5.96 Hz, 1H, CH-CO_2_H), 1.88 (m, 1H, CH), 0.75 (m, 6H, CH_3_). ^13^C NMR (DMSO-d_6_, 400MHz) δ: 172.7 (C = O), 141.7, 132.8, 129.4, 127.0 (aromatic carbons), 61.8, 30.9, 19.5, 18.4 (aliphatic carbons). HRMS-ESI (m/z): 258.1824 (M+H), calculated, 258.1827.

#### 1-(Benzenesulphonyl)-4-hydroxypyrrolidine-2-carboxylic acid (7o)

The amino acid was L-4-hydroxyproline, yield (3.3815 g, 99.99%), appearance, white powder, mp, 159.00°C. FTIR (KBr, cm^-1^): 3402 (OH), 2993, 2955 (C-H aliphatic), 1714 (C = O), 1589, 1484, 1450 (C = C), 1385, 1353 (2S = O), 1195, 1158 (SO_2_NH), 1158, 1100, 1070 (C-N, C-O). ^1^H NMR (DMSO-d_6_, 400MHz) δ: 7.77 (d, J = 7.32 Hz, 2H, ArH), 7.63 (t, J = 7.36 Hz, 1H, ArH), 7.56 (t, J = 7.80 Hz, 2H, ArH), 4.18 (s, 1H, OH), 4.04 (t, J = 7.80 Hz, 1H, CH-CO_2_H), 3.43 (m, 1H, CHOH), 3.09 (d, J = 11.00 Hz, 2H, CH_2_-N), 1.92 (m, 2H, CH_2_). ^13^C NMR (DMSO-d_6_, 400MHz) δ: 173.8 (C = O), 137.8, 133.5, 129.6, 127.9 (aromatic carbons), 68.9, 60.2, 56.8, 31.2 (aliphatic carbons). HRMS-ESI (m/z): 271.0518 (M^+^), calculated, 271.0514.

#### 1-(Benzenesulphonyl)-pyrrolidine-2-carboxylic acid (7p)

The amino acid was L-proline, yield (3.1911 g, 100%), appearance, yellowish oil. FTIR (KBr, cm^-1^): 3066 (C-H aromatic), 2983, 2884 (C-H aliphatic), 1730 (C = O), 1627, 1447 (C = C), 1343, 1292 (2S = O), 1199, 1161 (SO_2_NH), 1095, 1073, 1016 (C-N, C-O). ^1^H NMR (DMSO-d_6_, 400MHz) δ: 7.80 (m, 2H, ArH), 7.65 (m, 1H, ArH), 7.58 (m, 2H, ArH), 4.07 (dd, J = 4.60, 4.60 Hz, 1H, CH-CO_2_H), 3.31 (m, 1H, CH_a_ of CH_2_N), 3.11 (m, 1H, CH_b_ of CH_2_N), 1.78 (m, 3H), 1.50 (m, 1H). ^13^C NMR (DMSO-d_6_, 400MHz) δ: 173.7 (C = O), 137.8, 133.6, 129.9, 127.6 (aromatic carbons), 60.9 48.9, 30.9, 24.7 (aliphatic carbons). HRMS-ESI (m/z): 256.1679 (M+H), calculated 256.1680.

#### 2-(4-Nitrophenylsulphonamido)acetic acid (7q)

The amino acid was glycine, yield (2.2152 g, 94.59%), appearance, off-white powder, mp, 171.9–172.30°C. FTIR (KBr, cm^-1^): 3417 (OH of CO_2_H), 3297 (NH), 3108, 3070 (C-H aromatic), 1732 (C = O), 1605, 1482 (C = C), 1529, 1424 (N-O), 1354, 1333 (2S = O), 1240, 1165 (SO_2_NH), 1100, 1087, 1014 (C-N, C-O). ^1^H NMR (DMSO-d_6_, 400 MHz) δ: 8.42 (t, J = 4.00 Hz, 1H, NH), 8.35 (d, J = 9.15 Hz, 2H, ArH), 8.01 (d, J = 8.6 Hz, 2H, ArH), 3.66 (d, J = 4.00 Hz, 2H, CH_2_). ^13^C NMR (DMSO-d_6_, 400 MHz) δ: 170.7 (C = O), 150.0, 147.0, 128.7, 124.9 (aromatic carbons), 44.2 (aliphatic carbon). HRMS-ESI (m/z): 261.0261 (M+H), Calculated 261.0265.

#### 2-(4-Nitrophenylsulphonamido)-3-phenylpropanoic acid (7r)

The amino acid was L-phenylalanine, yield (3.1530 g, 99.99%), appearance, off-white powder, mp, 100.10–100.40°C. FTIR (KBr, cm^-1^): 3417 (OH of CO_2_H), 3253 (NH), 3182, 3111 (C-H aromatic), 1753 (C = O), 1607, 1456, 1435 (C = C), 1525, 1403 (N-O), 1347, 1311 (2S = O), 1164, 1124 (SO_2_NH), 1103, 1093 (C-N, C-O). ^1^H NMR (DMSO-d_6_, 400 MHz) δ: 8.68 (d, J = 8.6 Hz, 1H, NH), 8.16 (d, J = 9.15 Hz, 2H, ArH), 7.70 (d, J = 8.6 Hz, 2H, ArH), 7.08 (m, 5H, ArH), 3.93 (ddd, J = 5.2, 4.6, 5.15 Hz, 1H, CH-CH_2_), 2.95 (dd, J = 5.15, 5.15 Hz, 1H, CH_a_ of CH_2_), 2.67 (dd, J = 9.75, 9.75 Hz, 1H, CH_b_ of CH_2_). ^13^C NMR (DMSO-d_6_, 400 MHz) δ: 172.7 (C = O), 149.7, 147.2, 137.2, 129.7, 128.6, 128.2, 126.9, 124.7 (aromatic carbons), 58.2, 38.1 (aliphatic carbons). HRMS-ESI (m/z): 368.2347 (M+NH_4_)^+^, calculated 368.2341.

#### 3-(1H-Indol-2-yl)-2-(4-nitrophenylsulphonamido)propanoic acid (7s)

The amino acid was L-tryptophan, yield (3.4253 g, 97.94%), appearance, pale yellow powder, mp, 226.90–227.20°C. FTIR (KBr, cm^-1^): 3448 (OH of CO_2_H), 3380, 3295 (2NH), 3108 (C-H of aromatic), 1710 (C = O), 1607, 1459 (C = C), 1525, 1424 (N-O), 1348, 1332 (2S = O), 1167, 1119 (SO_2_NH), 1092, 1014 (C-N, C-O). ^1^H NMR (DMSO-d_6_, 400 MHz) δ: 10.66 (s, 1H, NH, indole), 8.60 (d, J = 8.00 Hz, 1H, NH), 7.85 (m, 2H, ArH), 7.48 (m, 2H, ArH), 7.23 (d, J = 7.45 Hz, 1H, ArH), 7.04 (d, J = 8.00 Hz, 1H, ArH), 6.99 (s, 1H, ArH), 6.85 (m, 2H, ArH), 3.89 (ddd, J = 4.00, 2.85, 4.6 Hz, 1H, CH-CO_2_H), 3.04 (dd, J = 4.6, 4.6 Hz, 1H, CH_a_ of CH_2_), 2.79 (dd, J = 10.3, 9.75 Hz, 1H, CH_b_ of CH_2_). ^13^C NMR (DMSO-d_6_, 400 MHz) δ: 173.3 (C = O), 148.9, 146.4, 136.5, 127.4, 126.9, 124.9, 123.8, 121.2, 118.8, 118.2, 111.7, 109.2 (aromatic carbons), 57.0, 28.4 (aliphatic carbons). HRMS-ESI (m/z): 390.0126 (M+H)^+^, calculated 390.0124.

#### 4-Methyl-2-nitrophenylsulphonamido)pentanoic acid (7t)

The amino acid was L-leucine, yield (2.8461 g, 99.98%), appearance, white powder, mp, 142.90–143.20°C. FTIR (KBr, cm^-1^): 3272 (NH), 3113, 3097, 3074 (C-H aromatic), 2934, 2878 (C-H aliphatic), 1714 (C = O), 1658, 1606, 1469 (C = C), 1531, 1417 (N-O), 1356, 1307 (2S = O), 1168, 1156 (SO_2_NH), 1108, 1091, 1010 (C-N, C-O). ^1^H NMR (DMSO-d_6_, 400 MHz) δ: 8.54 (d, J = 9.15 Hz, 1H, NH), 8.35 (d, J = 8.6 Hz, 2H, ArH), 7.98 (d, J = 8.6 Hz, 2H, ArH), 3.68 (m, 1H, CH-CO_2_H), 1.56 (m, 1H, CH(CH_3_)_2_), 1.38 (m, 2H, CH_2_), 0.76 (m, 6H, 2CH_3_). ^13^C NMR (DMSO-d_6_, 400 MHz) δ: 173.4 (C = O), 149.9, 147.3, 128.7, 124.9 (aromatic carbons), 54.7, 41.2, 24.5, 23.1, 21.4 (aliphatic carbons). HRMS-ESI (m/z): 315.1265 (M-H)^-^, calculated 315.1266.

#### 3-Methyl-2-(4-nitrophenylsulphonamido)pentanoic acid (7u)

The amino acid was L-isoleucine, yield (2.7894 g, 97.97%), appearance, white powder, mp, 131.40–131.70°C. FTIR (KBr, cm^-1^): 3271 (NH), 3110, 2967 (C-H aromatic), 2879, 2858 (C-H aliphatic), 1705 (C = O), 1647, 1607, 1472 (C = C), 1528, 1428 (N-O), 1361, 1351 (2S = O), 1175, 1142 (SO_2_NH), 1092, 1011 (C-N, C-O). ^1^H NMR (DMSO-d_6_, 400 MHz) δ: 8.42 (d, J = 9.15 Hz, 1H, NH), 8.35 (d, J = 8.60 Hz, 2H, ArH), 7.99 (d, J = 8.55 Hz, 2H, ArH), 3.61 (t, J = 6.00 Hz, 1H, CH-CO_2_H), 1.67 (m, 1H, CH), 1.32 (m, 1H, CH_a_ of CH_2_), 1.07 (m, 1H, CH_b_ of CH_2_), 0.75 (m, 6H, 2CH_3_). ^13^C NMR (DMSO-d_6_, 400 MHz) δ: 172.4 (C = O), 149.9, 147.2, 128.7, 124.8 (aromatic carbons), 60.9, 37.3, 24.9, 15.9, 11.5 (aliphatic carbons). HRMS-ESI (m/z): 317.2387 (M+H)^+^, calculated 317.2386.

#### 3-Methyl-2-(4-nitrophenylsulphonamido)butanoic acid (7v)

The amino acid was L-valine, yield (2.7200 g, 99.97%), appearance, white needles, mp, 182.60–182.90°C. FTIR (KBr, cm^-1^): 3415 (OH CO_2_H), 3278 (NH), 3113, 2969 (C-H aromatic), 2931, 2873 (C-H aliphatic), 1711 (C = O), 1638, 1607, 1411 (C = C), 1532, 1463 (N-O), 1356, 1312 (2S = O), 1169, 1146 (SO_2_NH), 1107, 1091, 1062, 1013 (C-N, C-O). ^1^H NMR (DMSO-d_6_, 400 MHz) δ: 8.4367–8.4184 (d, J = 9.15 Hz, 1H, NH), 8.3577–8.3405 (d, J = 8.6 Hz, 2H, ArH), 7.9992–7.9809 (d, J = 9.15 Hz, 2H, ArH), 3.5845–3.5536 (dd, J = 5.7, 6.3 Hz, 1H, CH-CO_2_H), 1.9756–1.9091 (m, 1H, CH (CH_3_)_2_), 0.8063–0.7559 (m, 6H, 2CH_3_). ^13^C NMR (DMSO-d_6_, 400 MHz) δ: 172.4 (C = O), 149.9, 147.3, 128.7, 124.8 (aromatic carbons), 61.9, 30.8, 19.6, 18.3 (aliphatic carbons). HRMS-ESI (m/z): 320.0579 (M+NH_4_), calculated, 320.0573.

#### 4-Hydroxy-1-(4-nitrophenylsulphonyl)pyrrolidine-2-carboxylic acid (7w)

The amino acid was L-hydroxyproline, yield (2.8459 g, 99.97%), appearance, white needles, mp, 199.8–200.20°C. FTIR (KBr, cm^-1^): 3491 (OH), 3416 (OH of CO_2_H), 3117, 3024 (C-H aromatic), 2966, 2767 (C-H aliphatic), 1750 (C = O), 1638, 1608, 1457 (C = C), 1527, 1427 (N-O), 1359, 1331 (2S = O), 1184, 1164 (SO_2_N), 1136, 1112, 1090, 1015, 1005 (C-N, C-O). ^1^H NMR (DMSO-d_6_, 500 MHz) δ: 8.36 (d, J = 9.15 Hz, 2H, ArH), 8.03 (d, J = 8.60 Hz, 2H, ArH), 4.17 (s, 1H, OH), 4.11 (t, J = 8.6 Hz, 1H, CH-CO_2_H), 3.45 (dd, J = 3.45, 3.40 Hz, 1H, CHOH), 3.20 (d, J = 10.90 Hz, 2H, CH_2_N), 2.02 (m, 1H, CH_a_, CH_2_), 1.91 (m, 1H, CH_b_ of CH_2_). ^13^C NMR (DMSO-d_6_, 500 MHz) δ: 173.5, 150.3, 143.6, 129.5, 124.9 (aromatic carbons), 69.0, 60.4, 57.1 (aliphatic carbons). HRMS-ESI (m/z): 303.2987 (M+H)^+^, calculated 303.2984.

#### 1-(4-Nitrophenylsulphonyl)pyrrolidine-2-carboxylic acid (7x)

The amino acid was L-proline, yield (2.6051 g, 96.39%), appearance, off-white needles, mp, 149.90–150.20°C. FTIR (KBr, cm^-1^): 3415 (OH of CO_2_H), 3111 (C-H aromatic), 2899, 2648 (C-H aliphatic), 1723 (C = O), 1641, 1604, (C = C), 1533, 1447 (N-O), 1353, 1305 (2S = O), 1199, 1165 (SO_2_N), 1072, 1024, 1008 (C-N, C-O). ^1^H NMR (DMSO-d_6_, 500 MHz) δ: 8.38 (m, 2H, ArH), 8.06 (m, 2H, ArH), 4.18 (m, 1H, CH-CO_2_H), 3.37 (m, 1H, CH_a_ of CH_2_), 3.20 (m, 1H, CH_b_ of CH_2_), 1.96 (m, 1H, CH_a_ of CH_2_), 1.81 (m, 2H, CH_2_), 1.63 (m, 1H, CH). ^13^C NMR (DMSO-d_6_, 500 MHz) δ: 173.4 (C = O), 150.4, 143.8, 129.2, 125.1 (aromatic carbons), 61.1, 48.9, 30.9, 24.7 (aliphatic carbons). HRMS-ESI (m/z): 301.0144 (M+H)^+^, calculated, 301.0143.

### General procedure for the synthesis of N-benzoyl derivatives of benzenesulphonamides (9a-f, i-n and q-v)

Appropriate benzenesulphonamides **(7a-f, i-n** and **q-v**, 1.0 mmol) was dissolved in NaOH (10%, 10 mL) in a 50 mL round bottom flask and benzoyl chloride (**8**, 1.1 mmol, 0.2 mL) was added into the solution and stirred at room temperature. The reaction progress was monitored by TLC (3% MeOH/DCM) to the disappearance of the benzenesulphonamide spot. Upon completion, the solution was transferred into a beaker containing crushed ice and then acidified to pH 3 with concentrated hydrochloric acid. The solid was collected via suction filtration and transferred into a beaker containing CCl_4_ (10 mL) and covered with watch glass boiled for 10 min. The mixture was allowed to cool slightly and then filtered. The products **(9a-f, i**-**n** and **q-v**) obtained were washed with 10–20 mL CCl_4_ and dried over fused self-indicating silica gel in a desiccator.

#### {Benzoyl[(4-methylphenyl)sulfonyl]amino}acetic acid (9a)

Yield (0.3281 g, 98.49%), appearance, white powder, mp, 104.60–104.80°C, FTIR (KBr, cm^-1^): 3072 (C-H aromatic), 1729, 1687 (2C = O), 1601, 1583, 1453, 1423 (C = C), 1327, 1293 (2S = O), 1157, 1186 (SO_2_N), 1128, 1094, 1073, 1001 (C-N, C-O). ^1^H NMR (DMSO-d_6_, 500MHz) δ: 7.64 (d, J = 8.6 Hz, 2H, ArH), 7.50 (m, 5H, ArH), 7.33 (d, J = 8.05 Hz, 2H, ArH), 3.51 (d, J = 5.7 Hz, CH_2_), 2.34 (s, 3H, CH_3_). ^13^C NMR (DMSO-d_6_, 500MHz) δ: 170.8, 167.8 (2C = O), 143.1, 138.4, 133.4, 131.3, 130.0, 129.8, 129.1, 127.1 (aromatic carbons), 44.3, 21.5 (aliphatic carbons). HRMS-ESI (m/z): 333.0679 (M^+^), calculated, 333.0671.

#### 2-[N-(4-methylbenzenesulfonyl)-1-phenylformamido]-3-phenylpropanoic acid (9b)

Yield (0.4201 g, 99.31%), appearance, white powder, mp, 100.50–100.70°C, FTIR (KBr, cm^-1^): 3443 (OH of CO_2_H), 3072 (C-H aromatic), 1732, 1689 (2C = O), 1603, 1585, 1497, 1454, 1425 (C = C), 1344, 1327, (2S = O), 1187, 1168, 1158 (SO_2_N), 1128, 1090, 1027 (C-N, C-O). ^1^H NMR (DMSO-d_6_, 500MHz) δ: 7.91 (m, 3H, ArH), 7.59 (t, J = 7.45 Hz, 2H, ArH), 7.45 (m, 5H, ArH), 17 (m, 3H, ArH), 7.08 (d, J = 7.45 Hz, 1H, ArH), 3.81 (t, J = 6.30 Hz, 1H, CH-CO_2_H), 2.89 (dd, J = 5.75, 5.75 Hz, 1H, CH_a_ of CH_2_), 2.67 (dd, J = 9.15, 8.55 Hz, 1H, CH_b_ of CH_2_), 2.30 (s, 3H, CH_3_-Ar). ^13^C NMR (DMSO-d_6_, 500MHz) δ: 172.8, 167.9 (2C = O), 142.8, 138.7, 137.3, 133.4, 133.2, 131.3, 129.8, 129.7, 129.1, 128.7, 126.9, 126.8 (aromatic carbons), 57.9, 38.4, 21.5 (aliphatic carbons). HRMS-ESI (m/z): 424.1142 (M+H), calculated, 424.1140.

#### 3-(1H-indol-2-yl)-2-[N-(4-methylbenzenesulfonyl)-1-phenylformamido]propanoic acid (9c)

Yield (0.4609 g, 99.74%), appearance, white powder, mp, 108.60–108.90°C, FTIR (KBr, cm^-1^): 3335 (NH), 3072 (C-H aromatic), 1761, 1688 (2C = O), 1619, 1602, 1584, 1454, 1424 (C = C), 1326, 1292 (2S = O), 1178, 1160 (SO_2_N), 1128, 1073, 1027 (C-N, C-O). ^1^H NMR (DMSO-d_6_, 500MHz) δ: 10.73 (s, 1H, NH of indole), 7.91 (t, J = 7.45 Hz, 3H, ArH), 7.58 (t, J = 7.45 Hz, 2H, ArH), 7.45 (m, 5H, ArH), 7.25 (t, J = 8.60 Hz, 1H, ArH), 7.13 (d, J = 8.05 Hz, 1H, ArH), 7.01 (t, J = 7.45 Hz, 1H, ArH), 6.88 (t, J = 7.45 Hz, 1H, ArH), 3.84 (dd, J = 7.45, 8.05 Hz, 1H, ArH), 3.01 (dd, J = 6.30, 6.30 Hz, 1H, CH_a_ of CH_2_), 2.80 (dd, J = 8.05, 8.00 Hz, 1H, CH_b_ of CH_2_), 2.28 (s, 3H, CH_3_-Ar). ^13^C NMR (DMSO-d_6_, 500MHz) δ: 173.2, 167.8 (2C = O), 142.7, 138.5, 136.6, 133.4, 131.3, 129.8, 129.7, 129.1, 127.4, 126.8, 124.5, 121.3, 118.8, 118.3, 111.9, 109.4 (aromatic carbons), 57.1, 28.8, 21.5 (aliphatic carbons). HRMS-ESI (m/z): 480.2135 (M+NH_4_), calculated, 480.2139.

#### 4-Methyl-2-[N-(4-methylbenzenesulfonyl)-1-phenylformamido]pentanoic acid (9d)

Yield (0.3889 g, 99.92%), appearance, white powder, mp, 98.90–99.60°C, FTIR (KBr, cm^-1^): 3415 (OH of CO_2_H), 2968 (C-H aromatic), 2676 (C-H aliphatic), 1705, 1688 (2C = O), 1619, 1602, 1584, 1454, 1424 (C = C), 1327, 1292 (2S = O), 1161, 1128 (SO_2_N), 1091, 1073, 1027 (C-N, C-O). ^1^H NMR (DMSO-d_6_, 500MHz) δ: 7.91 (t, J = 6.90 Hz, 3H, ArH), 7.59 (m, 2H, ArH), 7.46 (t, J = 8.00 Hz, 3H, ArH), 7.31 (d, J = 8.00 Hz, 1H, ArH), 3.59 (dd, J = 8.05, 8.55 Hz, 1H, CH-CO_2_H), 1.52 (m, 1H, CH), 1.33 (m, 2H, CH_2_), 0.76 (d, J = 6.30 Hz, 3H, CH_3_), 0.65 (d, J = 6.30 Hz, 3H, CH_3_). ^13^C NMR (DMSO-d_6_, 500MHz) δ: 173.8, 167.8 (2C = O), 142.9, 138.9, 133.4, 131.3, 129.9, 129.8, 129.1, 127.0 (aromatic carbons), 54.5, 41.5, 24.4, 23.1, 21.6, 21.5 (aliphatic carbons). HRMS-ESI (m/z): 390.2354 (M+H), calculated, 390.2358.

#### 3-Methyl-2-[N-(4-methylbenzenesulfonyl)-1-phenylformamido]pentanoic acid (9e)

Yield (0.3890 g, 99.95%), mp, 98.40–99.90°C, FTIR (KBr, cm^-1^): 3415 (OH of CO_2_H), 3072, 2970 (C-H of aromatic), 1707, 1688 (2C = O), 1602, 1584, 1496, 1454, 1424 (C = C), 1328, 1292 (2S = O), 1186, 1161 (SO_2_N), 1128, 1092, 1073, 1027 (C-N, C-O). ^1^H NMR (DMSO-d_6_, 500MHz) δ: 7.89 (m, 3H, ArH), 7.59 (m, 2H, ArH), 7.46 (t, J = 8.00 Hz, 3H, ArH), 7.30 (d, J = 8.00 Hz, 1H, ArH), 3.49 (d, J = 2.3 Hz, 1H, CH-CO_2_H), 2.33 (s, 3H, CH_3_-Ar), 1.61 (m, 1H, CH), 1.31 (m, 1H, CH_a_ of CH_2_), 1.05 (m, 1H, CH_b_ of CH_2_), 0.72 (dt, J = 6.30, 6.90 Hz, 6H, 2CH_3_). ^13^C NMR (DMSO-d_6_, 500MHz) δ: 172.7, 167.8 (2C = O), 142.9, 138.9, 133.4, 131.3, 129.8, 129.4, 129.1, 127.1 (aromatic carbons), 60.5, 37.5, 24.9, 21.5, 15.9, 11.4 (aliphatic carbons). HRMS-ESI (m/z): 388.0894 (M-H), calculated, 388.0896.

#### 3-Methyl-2-[N-(4-methylbenzenesulfonyl)-1-phenylformamido]butanoic acid acid (9f)

Yield (0.372 g, 99.20%), appearance, white powder, mp, 99.90–100.20°C, FTIR (KBr, cm^-1^): 3415 (OH, CO_2_H), 2970 (C-H aliphatic), 1706, 1688 (2C = O), 1618, 1602, 1584, 1454, 1424 (C = C), 1328, 1292 (2S = O), 1185, 1161 (SO_2_N), 1127, 1087, 1073 (C-N, C-O). ^1^H NMR (DMSO-d_6_, 500MHz) δ: 7.91 (d, J = 7.45 Hz, 2H, ArH), 7.59 (m, 4H, ArH), 7.45 (t, J = 8.00 Hz, 2H, ArH), 7.30 (d, J = 8.00 Hz), 3.45 (d, J = 4.60 Hz, 1H, CH-CO_2_H), 1.88 (m, 1H, CH), 0.77 (m, 6H, CH_3_). ^13^C NMR (DMSO-d_6_, 500MHz) δ: 172.7, 167.9 (2C = O), 142.9, 138.9, 133.4, 131.3, 129.8, 129.3, 129.1, 127.1 (aromatic carbons), 61.7, 30.9, 21.5, 19.5, 18.4 (aliphatic carbons). HRMS-ESI (m/z): 375.1148 (M^+^), calculated, 375.1140.

#### 2-[N-(benzenesulfonyl)-1-phenylformamido]acetic acid (9i)

Yield (0.2999 g, 93.92%), appearance, white needles, mp, 121.60–121.90°C, FTIR (KBr, cm^-1^): 3073, 3011 (C-H aromatic), 2837 (C-H aliphatic), 1732, 1688 (2C = O), 1619, 1603, 1584, 1454, 1425 (C = C), 1327, 1293 (2S = O), 1186, 1128 (SO_2_N), 1101, 1073, 1027 (C-N, C-O). ^1^H NMR (DMSO-d_6_, 400MHz) δ: 7.90 (t, J = 8.20 Hz, 4H, ArH), 7.57 (m, 2H, ArH), 7.45 (t, J = 7.80 Hz, 4H, ArH), 3.54 (s, 2H, CH_2_). ^13^C NMR (DMSO-d_6_, 400MHz) δ: 174.7, 167.9 (2C = O) 154.1, 151.1, 133.4, 131.3, 129.8, 129.1, 119.4, 112.8 (eight aromatic carbons), 59.0 (aliphatic carbon). HRMS-ESI (m/z): 320.0516 (M+H), calculated, 320.0514.

#### 2-[N-(benzenesulphonyl)-1-phenylformamido]-3-phenylpropanoic acid (9j)

Yield (0.4090 g, 99.90%), appearance, white powder, mp, 94.10–94.70°C, FTIR (KBr, cm^-1^): 3342 (OH of COOH), 3073, 3029 (C-H aromatic), 2968 (C-H aliphatic), 1735, 1688 (C = O), 1603, 1584, 1496, 1453, 1425 (C = C), 1376, 1348 (2S = O), 1170, 1108 (SO_2_N), 1094, 1073, 1027, 1000 (C-N, C-O). ^1^H NMR (DMSO-d_6_, 400MHz) δ: 7.91 (d, J = 8.00 Hz, 4H, ArH), 7.58 (t, J = 7.45 Hz, 2H, ArH), 7.52 (t, J = 8.00 Hz, 1H, ArH), 7.45 (m, 4H, ArH), 7.39 (t, J = 8.00 Hz, 1H, ArH), 7.15 (m, 2H, ArH), 7.08 (d, J = 6.30 Hz, 1H, ArH), 3.84 (t, J = 5.75 Hz, 1H, CH-COOH), 2.90 (dd, J = 5.70, 5.70 Hz, 1H, CH_a_ of CH_2_), 2.67 (dd, J = 9.15, 9.15 Hz, 1H, CH_b_ of CH_2_). ^13^C NMR (DMSO-d_6_, 400MHz) δ: 172.8, 167.8 (2C = O), 141.5, 137.3, 133.4, 132.6, 131.3, 129.8, 129.7, 129.4, 129.1, 128.7, 127.1, 126.8 (twelve aromatic carbons), 57.9, 38.3 (aliphatic carbons). HRMS-ESI (m/z): 410.0988 (M+H), calculated 410.0983.

#### 2-[N-(benzenesulfonyl)-1-phenylformamido]-3-(1H-indol-2-yl)propanoic acid (9k)

Yield (0.4480 g, 99.89%), appearance, white powder, mp, 110.90–111.20°C, FTIR (KBr, cm^-1^): 3385 (OH of COOH), 3305 (NH of indole), 3072 (C-H of aromatic), 2839 (C-H aliphatic), 1747, 1687 (2C = O), 1602, 1584, 1454, 1425 (C = C), 1339, 1292 (2S = O), 1162, 1129 (SO_2_N), 1083, 1027 (C-O, C-N). ^1^H NMR (DMSO-d_6_, 500MHz) δ: 10.79 (s, 1H, NH), 7.91 (m, 3H, ArH), 7.57 (m, 2H, ArH), 7.46 (m, 3H, ArH), 7.35 (t, J = 7.45 Hz, 2H, ArH), 7.26 (m, 2H, ArH), 7.01 (t, J = 6.85 Hz, 2H, ArH), 6.90 (t, J = 7.45 Hz, 1H, ArH), 3.87 (dd, J = 7.45, 8.05 Hz, 1H, CH-COOH), 3.02 (dd, J = 6.85, 6.30 Hz, 1H, CH_a_ of CH_2_), 2.82 (dd, J = 7.45, 7.45 Hz, 1H, CH_b_ of CH_2_). ^13^C NMR (DMSO-d_6_, 500MHz) δ: 173.1, 167.9 (2C = O), 141.4, 136.6, 133.4, 132.6, 131.3, 129.8, 129.2, 129.1, 127.5, 126.7, 124.5, 121.4, 118.9, 118.4, 111.9, 109.4 (sixteen aromatic carbons), 57.2, 28.8 (aromatic carbons). HRMS-ESI (m/z): 449.1096 (M+H), calculated, 449.1093.

#### 2-[N-(benzenesulfonyl)-1-phenylformamido]-4-methylpentanoic acid (9l)

Yield (0.3659 g, 97.86%), appearance, white powder, mp, 90.40–90.60°C, FTIR (KBr, cm^-1^): 3072 (C-H aromatic), 2959, 2874, 2838 (C-H aliphatic), 1721, 1687 (2C = O), 1603, 1584, 1454, 1426 (C = C), 1327, 1292 (2S = O), 1186, 1128 (SO_2_N), 1093, 1074, 1027 (C-N, C-O). ^1^H NMR (DMSO-d_6_, 500MHz) δ: 7.91 (t, J = 8.60 Hz, 3H, ArH), 7.74 (d, J = 7.45 Hz, 1H, ArH), 7.58 (m, 2H, ArH), 7.52 (t, J = 8.05 Hz, ArH), 7.46 (t, J = 8.00 Hz, 3H, ArH), 3.61 (m, 1H, CH-COOH), 1.49 (m, 1H, CH), 1.34 (m, 2H, CH_2_), 0.72 (m, 6H, CH_3_). ^13^C NMR (DMSO-d_6_, 400MHz) δ: 173.8, 167.9 (2C = O), 141.6, 133.4, 132.8, 131.3, 129.8, 129.5, 129.1, 126.9 (eight aromatic carbons), 54.5, 41.4, 24.3, 23.1, 21.5 (five aliphatic carbons). HRMS-ESI (m/z): 376.1143 (M+H), calculated, 376.1140.

#### 2-[N-(benzenesulfonyl)-1-phenylformamido]-3-methylpentanoic acid (9m)

Yield (0.3750 g, 99.89%), appearance, white powder, mp, 91.40–91.90°C, FTIR (KBr, cm^-1^): 3295 (OH of COOH), 3072 (C-H aromatic), 2969, 2883 (C-H aliphatic), 1723, 1698 (C = O), 1619, 1603, 1584, 1454, 1425 (C = C), 1327, 1293 (2S = O), 1169, 1129 (SO_2_N), 1092, 1074, 1027 (C-N, C-O). ^1^H NMR (DMSO-d_6_, 500MHz) δ: 7.89 (d, J = 7.15 Hz, 2H, ArH), 7.72 (d, J = 7.15 Hz, 1H, ArH), 7.55 (m, 2H, ArH), 7.47 (m, 5H, ArH), 3.50 (t, J = 6.30 Hz, 1H, CH-COOH), 1.59 (m, 1H, CH), 1.28 (m, 1H, CH_a_, CH_2_), 1.03 (m, 1H, CH_b_, CH_2_), 0.72 (d, J = 6.90 Hz, 3H, CH_3_-CH), 0.67 (t, J = 7.45 Hz, 3H, CH_3_-CH_2_). ^13^C NMR (DMSO-d_6_, 500MHz) δ: 172.7, 167.9 (2C = O), 141.5, 133.4, 132.8, 131.2, 129.8, 129.4, 129.1, 126.9 (eight aromatic carbons), 60.6, 37.4, 24.9, 15.8, 11.3 (five aliphatic carbons). HRMS-ESI (m/z): 376.1141 (M+H), calculated, 376.1140.

#### 2-[N-(benzenesulfonyl)-1-phenylformamido]-3-methylpentanoic acid (9n)

Yield (0.3050 g, 84.37%), appearance, white powder, mp, 104.60–104.90°C, FTIR (KBr, cm^-1^): 3302 (OH of COOH), 3073, 2970 (C-H aromatic), 2877 (C-H aliphatic), 1734, 1688 (2C = O), 1603, 1584, 1454, 1425 (C = C), 1327, 1293 (2S = O), 1169, 1129 (SO_2_N), 1128, 1093, 1074 (C-O, C-N). ^1^H NMR (DMSO-d_6_, 500MHz) δ: 7.91 (m, 3H, ArH), 7.74 (t, J = 7.45 Hz, 1H, ArH), 7.57 (m, 2H, ArH), 7.51 (d, J = 7.45 Hz, 1H, ArH), 7.46 (m, 3H, ArH), 3.49 (d, J = 6.30 Hz, 1H, CH-COOH), 1.88 (m, 1H, CH), 0.75 (m, 6H, 2CH_3_). ^13^C NMR (DMSO-d_6_, 500MHz) δ: 172.7, 167.9 (2C = O), 141.7, 133.4, 132.8, 131.3, 129.8, 129.4, 129.1, 127.0 (eight aromatic carbons), 61.8, 30.9, 19.5, 18.4 (aliphatic carbons). HRMS-ESI (m/z): 360.0984 (M-H), calculated, 360.0983.

#### 2-[N-(4-nitrobenzenesulfonyl)-1-phenylformamido]acetic acid (9q)

Yield (0.2126 g, 58.36%), appearance, white powder, mp, 135.00–135.60°C, FTIR (KBr, cm^-1^): 3310 (OH of COOH), 3073, 3009 (C-H aromatic), 2839 (C-H aliphatic), 1703, 1688 (2C = O), 1604, 1584, 1422 (C = C), 1527, 1454 (N-O), 1327, 1290 (2S = O), 1181, 1163 (SO_2_N), 1128, 1106, 1074 (C-N, C-O). ^1^H NMR (DMSO-d_6_, 500MHz) δ: 8.36 (d, 8.60 Hz, 2H, ArH), 8.01 (d, J = 8.60 Hz, 2H, ArH), 7.91 (d, J = 6.85 Hz, 2H, ArH), 7.58 (t, J = 7.45 Hz, 1H, ArH), 7.47 (t, J = 8.05 Hz, 2H, ArH), 3.67 (s, 2H, CH_2_). ^13^C NMR (DMSO-d_6_, 500MHz) δ: 170.7, 167.9 (2C = O), 150.0, 147.0, 133.4, 131.3, 129.8, 129.1, 128.7, 124.9 (aromatic carbons), 44.3 (CH_2_). HRMS-ESI (m/z): 363.0254 (M-H), calculated, 363.0251.

#### 2-[N-(4-nitrobenzenesulfonyl)-1-phenylformamido]-3-phenylpropanoic acid (9r)

Yield (0.4545 g, 100%), appearance, white powder, mp, 122.50–122.90°C, FTIR (KBr, cm^-1^): 3309 (OH of COOH), 3113, 3073, 3008 (C-H aromatic), 2840 (C-H aliphatic), 1703, 1685 (2C = O), 1604, 1584, 1454, 1421 (C = C), 1527 (N-O), 1327, 1289 (2S = O), 1181, 1163 (SO_2_N), 1128, 1106 1074, 1027 (C-N, C-O). ^1^H NMR (DMSO-d_6_, 400MHz) δ: 8.16 (m, 2H, ArH), 7.90 (t, J = 7.36 Hz, 2H, ArH), 7.70 (m, 2H, ArH), 7.59 (m, 1H, ArH), 7.46 (m, 2H, ArH), 7.07 (m, 5H, ArH), 3.92 (m, 1H, CH-CO_2_H), 2.95 (dd, J = 4.60, 4.56 Hz, 1H, CH_a_ of CH_2_), 2.65 (dd, J = 10.08, 10.08 Hz, 1H, CH_b_ of CH_2_). ^13^C NMR (DMSO-d_6_, 400MHz) δ: 172.7, 167.9 (2C = O), 149.6, 147.1, 137.2, 133.4, 131.3, 129.8, 129.7, 129.1, 128.6, 128.2, 126.9, 124.7 (twelve aromatic carbons), 58.2, 38.1 (two aliphatic carbons). HRMS-ESI (m/z): 455.1936 (M+H), calculated, 455.1932.

#### 3-(1H-indol-2-yl)-2-[N-(4-nitrobenzenesulfonyl)-1-phenylformamido]propanoic acid (9s)

Yield (0.4932 g, 99.64%), appearance, white powder, mp, 153.30–153.70°C, FTIR (KBr, cm^-1^): 3448 (OH of COOH), 3294 (NH of indole), 3072 (C-H aromatic), 1705, 1688 (C = O), 1605, 1584, 1454, 1425 (C = C), 1525 (N-O), 1327, 1292 (2S = O), 1179, 1167 (SO_2_N), 1129, 1092, 1073, 1027 (C-N, C-O). ^1^H NMR (DMSO-d_6_, 400MHz) δ: 10.68 (s, 1H, NH of indole), 7.91 (m, 3H, ArH), 7.84 (m, 1H, ArH), 7.58 (m, 2H, ArH), 7.46 (m, 3H, ArH), 7.22 (d, J = 7.80 Hz, 1H, ArH), 7.03 (d, J = 7.80 Hz, 1H, ArH), 6.98 (d, J = 2.32 Hz, 1H, ArH), 6.84 (m, 2H, ArH), 3.88 (m, 1H, CH-COOH), 3.03 (dd, J = 4.12, 4.12 Hz, 1H, CH_a_ of CH_2_), 2.77 (dd, J = 10.08, 10.56 Hz, 1H, CH_b_ of CH_2_). ^13^C NMR (DMSO-d_6_, 400MHz) δ: 173.3, 167.8 (2C = O), 148.9, 144.7, 138.9, 136.5, 133.4, 132.9, 131.3, 129.8, 129.1, 127.4, 124.9, 123.8, 122.4, 118.8, 118.2, 111.7 (sixteen aromatic carbons), 46.4, 36.8 (two aliphatic carbons). HRMS-ESI (m/z): 494.0945 (M+H), calculated 494.0943.

#### 3-Methyl-2-[N-(4-nitrobenzenesulfonyl)-1-phenylformamido]pentanoic acid (9t)

Yield (0.4201 g, 99.93%), appearance, white powder, mp, 110.40–110.60°C, FTIR (KBr, cm^-1^): 3293 (OH of COOH), 3117, 3072 (C-H aromatic), 2968, 2882 (C-H aliphatic), 1730, 1691 (2C = O), 1619, 1604, 1454, 1410 (C = C), 1531 (N-O), 1349, 1310 (2S = O), 1182, 1129 (SO_2_N), 1105, 1073, 1027, 1012 (C-N, C-O). ^1^H NMR (DMSO-d_6_, 400MHz) δ: 8.34 (d, J = 8.68 Hz, 2H, ArH), 7.98 (d, J = 8.72 Hz, 2H, ArH), 7.90 (t, J = 7.32 Hz, 2H, ArH), 7.57 (t, J = 7.32 Hz, 1H, ArH), 7.46 (t, J = 7.72 Hz, 2H, ArH), 3.60 (d, J = 2.28 Hz, CH-COOH), 1.67 (m, 1H, CH), 1.31 (m, 1H, CH_a_ of CH_2_), 1.12 (m, 1H, CH_b_ of CH_2_), 0.74 (m, 6H, 2CH_3_). ^13^C NMR (DMSO-d_6_, 400MHz) δ: 172.4, 167.8 (2C = O), 149.9, 147.2, 133.4, 131.3, 129.8, 129.1, 128.7, 124.8 (eight aromatic carbons) 60.9, 37.3, 24.8, 15.9, 11.5 (five aliphatic carbons). HRMS-ESI (m/z): 420.0995 (M^+^), calculated, 420.0991.

#### 4-Methyl-2-[N-(4-nitrobenzenesulfonyl)-1-phenylformamido]pentanoic acid (9u)

Yield (0.4204 g, 100%), appearance, white powder, mp, 116.30–116.60°C, FTIR (KBr, cm^-1^):3412 (OH of COOH), 2960 (C-H aliphatic), 1721, 1689 (2C = O), 1603, 1584, 1454, 1410 (C = C), 1531 (N-O), 1348, 1323 (2S = O), 1177, 1149 (SO_2_N), 1126, 1091, 1074, 1026 (C-N, C-O). ^1^H NMR (DMSO-d_6_, 400MHz) δ: 8.35 (d, J = 8.72 Hz, 2H, ArH), 7.97 (d, J = 9.16 Hz, 2H, ArH), 7.90 (t, J = 6.88 Hz, 2H, ArH), 7.58 (t, J = 7.36 Hz, 1H, ArH), 7.46 (m, 2H, ArH), 3.73 (t, J = 5.48 Hz, 1H, CH-COOH), 1.55 (m, 1H, CH_a_ of CH_2_), 1.38 (m, 1H, CH_b_ of CH_2_), 0.79 (d, J = 6.44 Hz, 3H, CH_3_), 0.70 (d, J = 6.40 Hz, 3H, CH_3_). ^13^C NMR (DMSO-d_6_, 400MHz) δ: 173.4, 167.9 (2C = O), 149.9, 147.3, 133.4, 131.3, 129.8, 129.1, 128.7, 124.9 (eight aromatic carbons), 54.7, 41.2, 24.4, 23.1, 21.4 (five aliphatic carbons). HRMS-ESI (m/z): 420.0999 (M^+^), calculated, 420.0991.

#### 3-Methyl-2-[N-(4-nitrobenzenesulfonyl)-1-phenylformamido]butanoic acid (9v)

Yield (0.4063 g, 99.98%), appearance, white powder, mp, 116.10–117.20°C, FTIR (KBr, cm^-1^): 3277 (OH of COOH), 3112, 3073 (C-H aromatic), 2969, 2874 (C-H aliphatic), 1710, 1687 (2C = O), 1606, 1584, 1454, 1424 (C = C), 1532 (N-O), 1356, 1327 (2S = O), 1169, 1146 (SO_2_N), 1129, 1091, 1063, 1027, 1013, 1000 (C-N, C-O). ^1^H NMR (DMSO-d_6_, 400MHz) δ: 8.34 (d, J = 9.16 Hz, 2H, ArH), 7.95 (d, J = 8.72 Hz, 2H, ArH), 7.90 (t, J = 6.88 Hz, 2H, ArH), 7.58 (t, J = 7.32 Hz, 2H, ArH), 7.45 (m, 2H, ArH), 3.57 (d, J = 2.76Hz, 1H, CH-COOH), 1.94 (m, 1H, CH), 0.77 (m, 6H, 2CH_3_). ^13^C NMR (DMSO-d_6_, 400MHz) δ: 172.4, 167.9 (2C = O), 149.9, 147.3, 133.4, 131.3, 129.8, 129.1, 128.7, 124.8 (eight aromatic carbons), 61.9, 30.8, 19.6, 18.3 (four aliphatic carbons). HRMS-ESI (m/z): 405.0732 (M-H), calculated, 405.0731.

### Boric acid catalysed direct amidation of unactivated carboxylic acid and 3-aminoquinoline

To a suspension of *N*-benzoyl-substituted-benzenesulphonamides (**9a-f, i-n, q-v, 7g-h, o-p** and **w-x**) (1.0 mmol) (1.0 mmol) in dry toluene (40 mL) equipped with Dean-Stark apparatus for azeotropic removal of water, was added 3-aminoquinoline (1.0 mmol) and boric acid (0.1 mmol) at room temperature and refluxed for 8 h. Upon completion (as monitored by TLC), the amide products were precipitated out from the reaction mixture by adding 40 mL *n*-hexane. The carboxamides were obtained via suction filtration, washed with *n*-hexane and dried over fused silica gel or concentrated using rotary evaporator and dried over vacuum in the case of oily products.

#### 2-[N-(4-methylbenzenesulfonyl)-1-phenylformamido]-N-(quinolin-3-yl)acetamide (11a)

Yield (0.4489 g, 99.43%), brownish oil, FTIR (KBr, cm^-1^): 3390 (NH), 3059 (C-H aromatic), 1702, 1688 (C = O), 1627 (C = N), 1602, 1584, 1493, 1451 (C = C), 1351, 1317 (2S = O), 1158, 1120 (SO_2_N), 1094, 1071, 1026, 1001 (C-N). ^1^H NMR (DMSO-d_6_, 400MHz) δ: 8.44 (s, 1H, NH), 7.91 (d, J = 7.76 Hz, 3H, ArH), 7.74 (d, J = 8.24 Hz, 1H, ArH), 7.64 (d, J = 7.80 Hz, 1H, ArH), 7.55 (t, J = 6.40 Hz, 3H, ArH), 7.43 (m, 3H, ArH), 7.27 (m, 3H, ArH), 7.12 (m, 1H, ArH), 3.54 (s, 2H, CH_2_), 2.28 (m, 3H, CH_3_). ^13^C NMR (DMSO-d_6_, 500MHz) δ: 170.8, 167.9 (C = O), 143.8, 143.2, 142.9, 141.4, 138.3, 133.4, 131.3, 130.0, 129.8, 129.4, 129.1, 128.9, 128.7, 127.5, 127.0, 126.1, 125.8, 124.6, 112.3 (nineteen aromatic carbons), 44.3, 21.4 (two aliphatic carbons). HRMS-ESI (m/z): 460.1265 (M+H), calculated, 460.1263.

#### 2-[N-(4-methylbenzenesulfonyl)-1-phenylformamido]-3-phenyl-N-(quinolin-3-yl)propanamide (11b)

Yield (0.5401 g, 98.34%), brownish oil, FTIR (KBr, cm^-1^): 3452 (NH), 3021 (C-H aromatic), 1682, 1659 (C = O), 1630 (C = N), 1601, 1497 (C = C), 1385, 1301 (2S = O), 1156, 1143 (SO_2_N), 1092 (C-N). ^1^H NMR (DMSO-d_6_, 400MHz) δ: 8.40 (s, 1H, ArH), 8.13 (d, J = 9.16 Hz, 1H, ArH), 7.91 (d, J = 7.32 Hz, 1H, ArH), 7.72 (d, J = 8.24 Hz, 1H, ArH), 7.55 (t, J = 6.88 Hz, 1H, ArH), 7.44 (m, 3H, ArH), 7.30 (m, 3H, ArH), 7.16 (m, 5H, ArH), 7.08 (m, 4H, ArH), 6.55 (s, 1H, NH), 3.81 (m, 1H, CH-C = O), 2.88 (dd, J = 5.52, 5.96 Hz, 1H, CH_a_ of CH_2_), 2.65 (dd, J = 9.16, 9.20 Hz, 1H, CH_b_ of CH_2_), 2.26 (m, 3H, CH_3_). ^13^C NMR (DMSO-d_6_, 400MHz) δ: 172.8, 167.9 (C = O), 143.9, 142.9, 142.8, 141.4, 138.7, 137.9, 137.3, 133.4, 129.9, 129.8, 129.7, 129.4, 128.9, 128.7, 128.7, 126.9, 126.8, 126.1, 125.8, 124.4, 112.0 (twenty one aromatic carbons), 57.9, 38.4, 21.5 (three aliphatic carbons). HRMS-ESI (m/z): 548.1169 (M-H), calculated, 548.1165.

#### 3-(1H-indol-2-yl)-2-[N-(4-methylbenzenesulfonyl)-1-phenylformamido]-N-(quinolin-3-yl)propanamide (11c)

Yield (0.5792 g, 98.47%), appearance, brown powder, mp, 88.10–88.80°C, FTIR (KBr, cm^-1^): 3413, 3373 (2NH), 3058 (C-H aromatic), 2922 (C-H aliphatic), 1702, 1698 (C = O), 1622 (C = N), 1597, 1494, 1457 (C = C), 1353, 1319 (2S = O), 1185, 1157 (SO_2_N), 1091, 1011 (C-N). ^1^H NMR (DMSO-d_6_, 400MHz) δ: 10.74 (s, 1H, NH of indole), 8.40 (s, 1H, ArH), 8.08 (d, J = 8.24 Hz, 1H, ArH), 7.91 (d, J = 6.88 Hz, 1H, ArH), 7.72 (d, J = 8.28 Hz, 1H, ArH), 7.56 (m, 1H, ArH), 7.44 (m, 3H, ArH), 7.26 (m, 5H, ArH), 7.11 (m, 4H, ArH), 7.01 (m, 2H, ArH), 6.87 (t, J = 7.32 Hz, 1H, ArH), 3.83 (m, 1H, CH-C = O), 3.01 (dd, J = 6.44, 6.40 Hz, 1H, CH_a_ of CH_2_), 2.79 (dd, J = 7.80, 7.76 Hz, 1H, CH_b_ of CH_2_), 2.26 (m, 3H, CH_3_). ^13^C NMR (DMSO-d_6_, 400MHz) δ: 173.2, 163.9 (C = O), 143.9, 142.9, 142.7, 141.5, 138.5, 136.6, 129.9, 129.8, 129.6, 129.4, 129.0, 128.7, 127.4, 126.9, 126.8, 126.1, 125.8, 124.5, 124.4, 121.3, 118.8, 118.3, 111.9, 111.8, 109.4 (twenty five aromatic carbons), 57.1, 28.8, 21.5 (three aliphatic carbons). HRMS-ESI (m/z): 588.1834 (M^+^), calculated, 588.1831.

#### 4-Methyl-2-[N-(4-methylbenzenesulfonyl)-1-phenylformamido]-N-(quinolin-3-yl)pentanamide (11d)

Yield (0.5010 g, 97.24%), brownish oil, FTIR (KBr, cm^-1^): 3359 (NH), 3063 (C-H aromatic), 2957, 2871 (C-H aliphatic), 1700, 1692 (C = O), 1626 (C = N), 1602, 1584, 1495, 1452 (C = C), 1353, 1317 (2S = O), 1292, 1160 (SO_2_N), 1094, 1072, 1026 (C-N). ^1^H NMR (DMSO-d_6_, 400MHz) δ: 8.45 (s, 1H, ArH), 8.02 (d, J = 8.72 Hz, 1H, ArH), 7.92 (t, J = 6.88 Hz, 1H, ArH), 7.75 (d, J = 8.28 Hz, 1H, ArH), 7.62 (d, J = 8.24 Hz, 1H, ArH), 7.57 (d, J = 8.24 Hz, 1H, ArH), 7.45 (t, J = 7.80 Hz, 1H, ArH), 7.31 (m, 2H, ArH), 7.19 (m, 3H, ArH), 7.11 (m, 5H, ArH), 6.53 (s, 1H, NH), 3.61 (t, J = 5.96 Hz, 1H, CH-C = O), 1.35 (m, 1H, CH), 1.20 (m, 2H, CH_2_), 0.79 (m, 6H, 2CH_3_). ^13^C NMR (DMSO-d_6_, 400MHz) δ: 173.8, 167.9 (C = O), 143.5, 143.0, 142.9, 141.0, 138.9, 137.9, 133.4, 130.0, 129.9, 129.8, 129.4, 129.2, 129.1, 128.9, 128.7, 128.4, 127.1, 126.1, 125.8, 124.6, 112.5 (twenty one aromatic carbons), 54.5, 34.7, 23.1, 21.6, 14.5 (five aliphatic carbons). HRMS-ESI (m/z): 514.0101 (M-H), calculated, 514.0109.

#### 3-Methyl-2-[N-(4-methylbenzenesulfonyl)-1-phenylformamido]-N-(quinolin-3-yl) pentanamide (11e)

Yield (0.4998 g, 97.01%), brownish oil, FTIR (KBr, cm^-1^): 3363 (NH), 3063 (C-H aromatic), 2962, 2931, 2876 (C-H aliphatic), 1704, 1675 (C = O), 1627 (C = N), 1585, 1493, 1451 (C = C), 1317, 1304 (2S = O), 1258, 1159 (SO_2_N), 1094, 1071, 1025 (C-N). ^1^H NMR (DMSO-d_6_, 400MHz) δ: 8.41 (s, 1H, ArH), 7.91 (m, 3H, ArH), 7.72 (d, J = 8.24 Hz, 1H, ArH), 7.58 (m, 3H, ArH), 745 (m, 2H, ArH), 7.30 (m, 3H, ArH), 7.12 (m, 2H, ArH), 3.48 (d, J = 5.96 Hz, 1H, CH-C = O), 2.31 (s, 3H, CH_3_-Ar), 1.60 (m, 1H, CH), 1.32 (m, 1H, CH_a_ of CH_2_), 1.04 (m, 1H, CH_b_ of CH_2_), 0.70 (m, 6H, 2CH_3_). ^13^C NMR (DMSO-d_6_, 400MHz) δ: 172.7, 167.9 (C = O), 143.8, 142.9, 142.9, 141.3, 138.8, 133.4, 131.3, 130.0, 129.8, 129.4, 129.1, 128.9, 127.1, 126.9, 126.1, 124.5, 112.2 (seventeen aromatic carbons), 60.5, 37.4, 24.9, 21.5, 15.9, 11.4 (six aliphatic carbons). HRMS-ESI (m/z): 515.1889 (M^+^), calculated 515.1879.

#### 3-Methyl-2-[N-(4-methylbenzenesulfonyl)-1-phenylformamido]-N-(quinolin-3-yl)butanamide (11f)

Yield (0.4979 g, 99.34%), brownish oil, FTIR (KBr, cm^-1^): 3390 (NH), 3059 (C-H aromatic), 2965 (C-H aliphatic), 1704, 1668 (C = O), 1632 (C = N), 1603, 1493, 1451 (C = C), 1317, 1300 (2S = O), 1166, 1134 (SO_2_N), 1093, 1071, 1043, 1019 (C-N). ^1^H NMR (DMSO-d_6_, 400MHz) δ: 8.43 (s, 1H, ArH), 7.88 (t, J = 8.24 Hz, 2H, ArH), 7.75 (d, J = 8.24 Hz, 2H, ArH), 7.58 (m, 3H, ArH), 7.45 (t, J = 7.80 Hz, 1H, ArH), 7.33 (m, 5H, ArH), 7.18 (d, J = 2.32 Hz, 2H, ArH), 6.50 (s, 1H, NH), 3.78 (d, J = 3.20 Hz, 1H, CH-C = O), 2.28 (m, 3H, CH_3_-Ar), 1.87 (m, 1H, CH), 0.76 (m, 6H, 2CH_3_). ^13^C NMR (DMSO-d_6_, 400MHz) δ: 172.7, 167.9 (C = O), 159.3, 143.0, 142.9, 140.4, 138.9, 133.4, 131.3, 130.1, 129.8, 129.8, 129.1, 128.2, 127.3, 127.1, 126.2, 124.9, 113.1 (sixteen aromatic carbons), 61.7, 30.9, 21.5, 19.5, 18.4 (five aliphatic carbons). HRMS-ESI (m/z): 500.1604 (M-H), calculated, 500.1606.

#### 4-Hydroxy-1-(4-methylbenzenesulfonyl)-N-(quinolin-3-yl)pyrrolidine-2-carboxamide (11g)

Yield (0.4111 g, 100%), brownish oil, FTIR (KBr, cm^-1^): 3437 (OH), 3398 (NH), 2926 (C-H aliphatic), 1728 (C = O), 1631 (C = N), 1597, 1496, 1403 (C = C), 1332, 1305 (2S = O), 1198, 1156 (SO_2_N), 1092, 1013 (C-N). ^1^H NMR (DMSO-d_6_, 400MHz) δ: 8.39 (s, 1H, ArH), 7.72 (d, J = 8.24 Hz, 1H, ArH), 7.64 (d, J = 8.24 Hz, 2H, ArH), 7.55 (d, J = 8.24 Hz, 1H, ArH), 7.28 (m, 2H, ArH), 4.17 (s, 1H, OH), 4.01 (t, J = 4.24 Hz, 1H, CH-C = O), 3.41 (m, 1H, CH-OH), 3.04 (d, J = 5.32 Hz, 2H, CH_2_N), 2.35 (s, 3H, CH_3_), 1.90 (m, 2H, CH_2_). ^13^C NMR (DMSO-d_6_, 400MHz) δ: 173.8 (C = O), 143.9, 143.7, 134.9, 130.1, 129.4, 129.0, 128.7, 127.9, 126.9, 126.1, 125.9, 124.4, 111.9 (thirteen aromatic carbons), 68.9, 60.2, 56.8, 21.5 (four aliphatic carbons). HRMS-ESI (m/z): 411.1323 (M+H), calculated, 411.328.

#### 1-(4-Methylbenzenesulfonyl)-N-(quinolin-3-yl)pyrrolidine-2-carboxamide (11h)

Yield (0.3949 g, 99.95%), brownish oil, FTIR (KBr, cm^-1^): 3435 (NH), 2979 (C-H aliphatic), 1700 (C = O), 1626 (C = N), 1602, 1597, 1498, 1440 (C = C), 1338, 1304 (2S = O), 1197, 1158 (SO_2_N), 1094, 1013 (C-N). ^1^H NMR (DMSO-d_6_, 400MHz) δ: 8.44 (s, 1H, ArH), 7.73 (d, J = 8.24 Hz, 1H, ArH), 7.66 (d, J = 8.24 Hz, 2H, ArH), 7.55 (d, J = 7.76 Hz, 1H, ArH), 7.31 (m, 3H, ArH), 7.14 (m, 2H, ArH), 4.04 (t, J = 4.56 Hz, 1H, CH-C = O), 3.29 (m, 1H, CH_a_ of CH_2_N), 3.07 (m, 1H, CH_b_ of CH_2_N), 2.33 (s, 3H, CH_3_), 1.76 (m, 3H), 1.47 (m, 1H, CH). ^13^C NMR (DMSO-d_6_, 400MHz) δ: 173.7 (C = O), 143.6, 142.9, 141.1, 135.1, 130.4, 129.4, 128.7, 127.7, 127.1, 126.1, 125.8, 124.6, 112.4 (thirteen aromatic carbons), 60.9, 48.9, 30.9, 24.8, 21.5 (five aliphatic carbons). HRMS-ESI (m/z): 395.1307 (M^+^), calculated, 395.1304.

#### 2-[N-(benzenesulfonyl)-1-phenylformamido]-N-(quinolin-3-yl)acetamide (11i)

Yield (0.4001 g, 89.89%), brownish oil, FTIR (KBr, cm^-1^): 3391 (NH), 3071 (C-H aromatic), 2835 (C-H aliphatic), 1688 (C = O), 1688 (C = O), 1623 (C = N), 1603, 1453, 1424 (C = C), 1350, 1325 (2S = O), 1187, 1129 (SO_2_N), 1072, 1026 (C-N). ^1^H NMR (DMSO-d_6_, 500MHz) δ: 8.44 (s, 1H, ArH), 7.93 (d, J = 8.05 Hz, 3H, ArH), 7.74 (d, J = 8.10 Hz, 1H, ArH), 7.56 (t, J = 8.10 Hz, 2H, ArH), 7.46 (t, J = J = 8.05 Hz, 3H, ArH), 7.30 (m, 2H, ArH), 7.20 (t, J = 8.05 Hz, 2H, ArH), 7.11 (m, 2H, ArH), 2.25 (s, 3H, CH_3_). ^13^C NMR (DMSO-d_6_, 500MHz) δ: 167.9, 163.8 (C = O), 143.9, 142.9, 141.5, 137.9, 133.4, 131.3, 130.0, 129.8, 129.5, 129.4, 129.3, 129.1, 129.0, 128.7, 127.7, 126.9, 126.1, 125.8, 124.6, 124.4 (twenty aromatic carbons), 21.6 (aliphatic carbons). HRMS-ESI (m/z): 445.1112 (M^+^), calculated, 445.1096.

#### 2-[N-(benzenesulfonyl)-1-phenylformamido]-3-phenyl-N-(quinolin-3-yl)propanamide (11j)

Yield (0.5300 g, 98.95%), appearance, white powder, 112.70–112.90°C, FTIR (KBr, cm^-1^): 3389 (NH), 3066 (C-H aromatic), 1692, 1673 (C = O), 1623 (C = N), 1601, 1467 (C = C), 1343, 1321 (2S = O), 1178, 1154 (SO_2_N), 1092, 1053 (C-N). ^1^H NMR (DMSO-d_6_, 400MHz) δ: 8.47 (d, J = 2.72 Hz, 1H, ArH), 8.27 (d, J = 8.72 Hz, 1H, ArH), 7.79 (d, J = 8.24 Hz, 2H, ArH), 7.61 (m, 1H, ArH), 7.55 (m, 2H, ArH), 7.48 (m, 2H, ArH), 7.38 (t, J = 8.24 Hz, 3H, ArH), 7.33 (m, 1H, ArH), 7.31 (m, 1H, ArH), 7.23 (m, 1H, ArH), 7.16 (m, 5H, ArH), 7.08 (m, 2H, ArH), 3.87 (m, 1H, CH-C = O), 2.92 (dd, J = 5.48, 5.52 Hz, 1H, CH_a_ of CH_2_), 2.69 (dd, J = 9.16, 9.20 Hz, 1H, CH_a_ of CH_2_). ^13^C NMR (DMSO-d_6_, 400MHz) δ: 172.8, 167.9 (C = O), 142.8, 140.1, 137.7, 137.2, 133.4, 132.7, 131.3, 130.1, 129.8, 129.7, 129.4, 129.1, 128.7, 128.5, 127.9, 127.4, 127.1, 126.7, 126.2, 125.8, 125.1, 113.5 (twenty one aromatic carbons), 57.9, 38.4 (two aliphatic carbons). HRMS-ESI (m/z): 535.1667 (M^+^), calculated, 535.1666.

#### 2-[N-(benzenesulfonyl)-1-phenylformamido]-3-(1H-indol-2-yl)-N-(quinolin-3-yl)propanamide (11k)

Yield (0.5701 g, 99.22%), appearance, white powder, mp, 73.50–73.80°C, FTIR (KBr, cm^-1^): 3412, 3369 (2NH), 3002 (C-H aromatic), 2984 (C-H aliphatic), 1701, 1689 (C = O), 1622 (C = N), 1601, 1587, 1453 (C = C), 1342, 1309 (2S = O), 1185, 1134 (SO_2_N), 1089, 1045 (C-N). ^1^H NMR (DMSO-d_6_, 400MHz) δ: 10.76 (s, 1H, NH), 8.39 (d, J = 2.72 Hz, 1H, ArH), 8.18 (s, 1H, ArH), 7.71 (d, J = 8.24 Hz, 1H, ArH), 7.55 (m, 3H, ArH), 7.47 (m, 2H, ArH), 7.31 (m, 6H, ArH), 7.09 (d, J = 2.76 Hz, 1H, ArH), 7.01 (m, 2H, ArH), 6.90 (t, J = 7.34 Hz, 1H, ArH), 3.86 (m, 1H, CH-C = O), 3.02 (dd, J = 6.88, 6.44 Hz, 1H, CH_a_ of CH_2_), 2.81 (dd, J = 7.80, 7.80 Hz, 1H, CH_b_ of CH_2_). ^13^C NMR (DMSO-d_6_, 400MHz) δ: 173.1, 167.1 (C = O), 151.2 143.9, 142.9, 141.4, 136.6, 133.9, 133.5, 132.6, 130.0, 129.9, 129.8, 129.4, 129.2, 129.0, 128.7, 127.5, 126.9, 126.7, 126.1, 124.4, 121.4, 118.9, 118.4, 111.9, 109.4 (twenty five aromatic carbons), 57.2, 28.8 (two aliphatic carbons). HRMS-ESI (m/z): 573.2795 (M-H), calculated, 573.2796.

#### 2-[N-(benzenesulfonyl)-1-phenylformamido]-4-methyl-N-(quinolin-3-yl)pentanamide (11l)

Yield (0.4912 g, 98.00%), appearance, white powder, 132.40–132.80°C, FTIR (KBr, cm^-1^): 3362 (NH), 3062 (C-H aromatic), 2958 (C-H aliphatic), 1704, 1687 (C = O), 1628 (NO_2_), 1603, 1450 (C = C), 1353, 1315 (2S = O), 1260, 1163 (SO_2_N), 1094, 1071, 1025 (C-N). ^1^H NMR (DMSO-d_6_, 400MHz) δ: 8.40 (s, 1H, ArH), 8.11 (d, J = 8.72 Hz, 2H, ArH), 7.91 (d, J = 6.84 Hz, 2H, ArH), 7.71 (d, J = 1.84 Hz, 3H, ArH), 7.51 (m, 5H, ArH), 7.31 (m, 3H, ArH), 3.59 (m, 1H, CH-C = O), 1.50 (m, 1H, CH), 1.32 (m, 2H, CH_2_), 0.71 (m, 6H, CH_3_). ^13^C NMR (DMSO-d_6_, 400MHz) δ: 173.8, 162.7 (C = O), 143.9, 142.9, 141.6, 141.4, 138.7, 134.3, 133.4, 132.8, 129.8, 129.5, 129.1, 128.9, 126.9, 126.1, 124.4, 112.0, 100.0 (seventeen aromatic carbons), 54.5, 41.4, 24.4, 23.1 (four aliphatic carbons). HRMS-ESI (m/z): 500.1563 (M-H), calculated, 500.1568.

#### 2-[N-(benzenesulfonyl)-1-phenylformamido]-3-methyl-N-(quinolin-3-yl)pentanamide (11m)

Yield (0.4514 g, 90.06%), appearance, brown powder, 145.10–145.30°C, FTIR (KBr, cm^-1^): 3361 (NH), 3062 (C-H aromatic), 2933, 2877 (C-H aliphatic), 1704, 1687 (C = O), 1627 (C = N), 1603, 1584, 1500, 1449 (C = C), 1353, 1315 (2S = O), 1259, 1162 (SO_2_N), 1093, 1071, 1025 (C-N). ^1^H NMR (DMSO-d_6_, 400MHz) δ: 8.42 (d, J = 2.76 Hz, 1H, ArH), 7.91 (m, 2H, ArH), 7.74 (m, 2H, ArH), 7.51 (m, 5H, ArH), 7.29 (m, 3H, ArH), 7.19 (t, J = 7.32 Hz, 1H, ArH), 7.09 (m, 2H, ArH), 3.52 (d, J = 2.76 Hz, 1H, CH-C = O), 1.61 (m, 1H, CH), 1.30 (m, 1H, CH_a_ of CH_2_), 1.04 (m, 1H, CH_b_ of CH_2_), 0.71 (m, 6H, 2CH_3_). ^13^C NMR (DMSO-d_6_, 400MHz) δ: 172.7, 167.9 (C = O), 143.8, 142.9, 141.7, 141.4, 133.4, 132.8, 131.3, 130.0, 129.8, 129.4, 129.1, 128.9, 128.7, 127.0, 126.9, 126.1, 125.8, 124.5, 112.1 (nineteen aromatic carbons), 60.6, 37.4, 24.9, 15.9, 11.4 (five aliphatic carbons). HRMS-ESI (m/z): 501.1742 (M^+^), calculated, 501.1742.

#### 2-[N-(benzenesulfonyl)-1-phenylformamido]-3-methyl-N-(quinolin-3-yl)butanamide (11n)

Yield (0.4511 g, 92.59%), brownish oil, FTIR (KBr, cm^-1^): 3361 (NH), 3063 (C-H aromatic), 2966 (C-H aliphatic), 1703, 1664 (C = O), 1627 (C = N), 1603, 1584, 1498, 1450 (C = C), 1353, 1316 (2S = O), 1273, 1164 (SO_2_N), 1094, 1071, 1025, 1000 (C-N). ^1^H NMR (DMSO-d_6_, 400MHz) δ: 8.46 (s, 1H, ArH), 8.01 (m, 3H, ArH), 7.76 (t, J = 8.05 Hz, 2H, ArH), 7.50 (m, 5H, ArH), 7.30 (m, 3H, ArH), 7.19 (t, J = 8.05 Hz, 1H, ArH), 7.10 (m, 2H, ArH), 3.51 (d, J = 6.44 Hz, 1H, CH-C = O), 1.92 (m, 1H, CH), 0.76 (m, 6H, 2CH_3_). ^13^C NMR (DMSO-d_6_, 400MHz) δ: 172.8, 167.9 (C = O), 143.9, 142.9, 141.7, 141.5, 137.9, 133.4, 131.3, 129.8, 129.4, 129.4, 129.1, 128.9, 128.7, 127.0, 126.9, 126.1, 125.8, 124.5 (eighteen aromatic carbons), 61.8, 30.9, 19.5, 18.3 (four aliphatic carbons). HRMS-ESI (m/z): 486.1435 (M-H), calculated, 486.1437.

#### 1-(Benzenesulfonyl)-4-hydroxy-N-(quinolin-3-yl)pyrrolidine-2-carboxamide (11o)

Yield (0.3970 g, 99.89%), brownish oil, FTIR (KBr, cm^-1^): 3416 (OH), 3365 (NH), 3065 (C-H aromatic), 2974, 2879 (C-H aliphatic), 1701 (C = O), 1618 (C = N), 1601, 1447 (C = C), 1384, 1332 (2S = O), 1172, 1157 (SO_2_N), 1092, 1045 (C-N). ^1^H NMR (DMSO-d_6_, 400MHz) δ: 8.42 (s, 1H, ArH), 7.75 (m, 3H, ArH), 7.60 (m, 4H, ArH), 7.25 (m, 2H, ArH), 7.11 (s, 1H, ArH), 6.69 (s, 1H, NH), 4.18 (s, 1H, OH), 4.05 (t, J = 7.80 Hz, 1H, CH-C = O), 3.43 (m, 1H, CH-OH), 3.10 (d, J = 5.24 Hz, 2H, CH_2_N), 1.92 (m, 2H, CH_2_). ^13^C NMR (DMSO-d_6_, 400MHz) δ: 173.8 (C = O), 143.9, 141.5, 137.8, 133.5, 129.6, 129.4, 128.9, 128.7, 127.9, 126.9, 126.1, 125.8, 124.5, 112.1 (thirteen aromatic carbons), 68.9, 60.2, 56.9, 23.0 (four aliphatic carbons). HRMS-ESI (m/z): 398.1172 (M+H), calculated, 398.1176.

#### 1-(Benzenesulfonyl)-N-(quinolin-3-yl)pyrrolidine-2-carboxamide (11p)

Yield (0.3813 g, 99.98%), brownish oil, FTIR (KBr, cm^-1^): 3417 (NH), 3063 (C-H aromatic), 2978, 2877 (C-H aliphatic), 1725 (C = O), 1619 (C = N), 1499, 1446 (C = C), 1384, 1339 (2S = O), 1198, 1160 (SO_2_N), 1094, 1072, 1016 (C-N). ^1^H NMR (DMSO-d_6_, 500MHz) δ: 8.41 (s, 1H, ArH), 7.79 (t, J = 6.85 Hz, 2H, ArH), 7.73 (d, J = 8.00 Hz, 1H, ArH), 7.66 (t, J = 7.45 Hz, 1H, ArH), 7.58 (m, 3H, ArH), 7.30 (m, 1H, ArH), 7.21 (t, J = 7.45 Hz, 1H, ArH), 7.11 (m, 1H, ArH), 6.50 (s, 1H, NH), 4.07 (m, 1H, CH-C = O), 3.33 (m, 1H, CH_a_ of CH_2_N), 3.12 (m, 1H, CH_b_ of CH_2_N), 1.81 (m, 3H), 1.52 (m, 1H, CH).^13^C NMR (DMSO-d_6_, 500MHz) δ: 173.7 (C = O), 143.8, 142.9, 141.3, 138.0, 133.6, 129.9, 129.4, 128.9, 128.7, 127.6, 127.0, 126.1, 125.9, 124.5, 112.2 (fifteen aromatic carbons), 60.9, 48.9, 30.9, 24.8 (four aliphatic carbons). HRMS-ESI (m/z): 399.2098 (M+NH_4_), calculated, 399.2096.

#### 2-[N-(4-nitrobenzenesulfonyl)-1-phenylformamido]-N-(quinolin-3-yl)acetamide (11q)

Yield (0.4889 g, 99.76%), appearance, white powder, mp, 102.80–102.90°C, FTIR (KBr, cm^-1^): 3377 (NH), 3091 (C-N aromatic), 1745, 1672 (C = O), 1619 (C = N), 1601, 1437 (C = C), 1527 (NO_2_), 1350, 1306 (2S = O), 1256, 1161 (SO_2_N), 1092 (C-N). ^1^H NMR (DMSO-d_6_, 400MHz) δ: 8.70 (d, J = 9.16 Hz, 1H, ArH), 8.44 (m, 2H, ArH), 8.13 (m, 2H, ArH), 7.91 (m, 2H, ArH), 7.73 (m, 2H, ArH), 7.59 (m, 1H, ArH), 7.45 (m, 1H, ArH), 7.35 (m, 1H, ArH), 7.22 (m, 2H, ArH), 7.07 (m, 2H, ArH), 3.13 (s, 2H, CH_2_). ^13^C NMR (DMSO-d_6_, 400MHz) δ: 172.7, 167.9 (C = O), 147.1, 143.1, 142.7, 139.9, 130.1, 129.8, 129.7, 129.4, 129.1, 128.7, 128.6, 128.2, 127.8, 127.4, 126.2, 125.2, 124.7 (seventeen aromatic carbons), 58.2 (aliphatic carbons). HRMS-ESI (m/z): 490.1714 (M^+^), calculated, 490.1718.

#### 2-[N-(4-nitrobenzenesulfonyl)-1-phenylformamido]-3-phenyl-N-(quinolin-3-yl)propanamide (11r)

Yield (0.5529 g, 95.48%), brownish oil, FTIR (KBr, cm^-1^): 3359 (NH), 3063 (C-H aromatic), 1705, 1697 (C = O), 1602 (C = N), 1584, 1499, 1451 (C = C), 1350, 1315 (2S = O), 1165, 1118 (SO_2_N), 1088, 1071, 1025 (C-N). ^1^H NMR (DMSO-d_6_, 400MHz) δ: 8.44 (m, 1H, ArH), 8.33 (m, 1H, ArH), 8.15 (d, J = 9.16 Hz, 1H, ArH), 8.02 (d, J = 9.16 Hz, 1H, ArH), 7.91 (m, 3H, ArH), 7.72 (t, J = 7.32 Hz, 2H, ArH), 7.56 (m, 2H, ArH), 7.45 (m, 3H, ArH), 7.30 (m, 3H, ArH), 7.12 (d, J = 6.58 Hz, 2H, ArH), 7.07 (m, 1H, ArH), 3.95 (m, 1H, CH-C = O), 2.96 (dd, J = 5.04, 5.04 Hz, 1H, CH_a_ of CH_2_), 2.68 (dd, J = 10.08, 10.08 Hz, 1H, CH_b_ of CH_2_). ^13^C NMR (DMSO-d_6_, 400MHz) δ: 172.7, 167.9 (C = O), 143.8, 142.9, 141.3, 137.2, 133.4, 131.3, 130.0, 129.8, 129.7, 129.4, 129.1, 128.9, 128.7, 128.6, 128.2, 126.9, 126.9, 126.1, 124.9, 124.7, 124.5, 112.2 (twenty one aromatic carbons), 58.2, 38.1 (two aliphatic carbons). HRMS-ESI (m/z): 597.1860 (M+NH_4_), calculated, 597.1864.

#### 3-(1H-indol-2-yl)-2-[N-(4-nitrobenzenesulfonyl)-1-phenylformamido]-N-(quinolin-3-yl) propanamide (11s)

Yield (0.6089 g, 98.34%), appearance, white powder, 147.70–147.90°C, FTIR (KBr, cm^-1^): 3416, 3387 (NH), 3076 (C-H aromatic), 1710, 1684 (C = O), 1627 (C = N), 1607 (C = C), 1527 (NO_2_), 1350, 1312 (2S = O), 1164, 1142 (SO_2_N), 1093 (C-N). ^1^H NMR (DMSO-d_6_, 400MHz) δ: 10.69 (s, 1H, NH indole), 8.61 (d, J = 8.24 Hz, 1H, ArH), 8.41 (s, 1H, ArH), 8.05 (s, 1H, ArH), 7.87 (m, 2H, ArH), 7.72 (d, J = 8.24 Hz, 1H, ArH), 7.55 (d, J = 7.76 Hz, 1H, ArH), 7.46 (d, J = 8.68 Hz, 3H, ArH), 7.26 (m, 4H, ArH), 7.12 (m, 1H, ArH), 7.04 (d, J = 8.24 Hz, 1H, ArH), 6.99 (m, 1H, ArH), 6.84 (m, 3H, ArH), 6.48 (s, 1H, NH), 3.88 (m, 1H, CH-C = O), 3.03 (dd, J = 4.56, 4.12 Hz, 1H, CH_a_ of CH_2_), 2.78 (dd, J = 10.52, 10.08 Hz, 1H, CH_b_ of CH_2_). ^13^C NMR (DMSO-d_6_, 400MHz) δ: 173.3, 164.1 (C = O), 158.1, 148.9, 148.4, 146.4, 143.8, 142.9, 141.3, 136.5, 130.0, 129.8, 129.4, 128.9, 128.7, 127.4, 126.9, 126.1, 124.9, 124.4, 123.8, 121.2, 118.8, 118.2, 112.1, 111.7, 109.2 (twenty four aromatic carbons), 57.1, 28.4 (two aliphatic carbons). HRMS-ESI (m/z): 637.1603 (M+NH_4_), calculated, 637.1609.

#### 4-Methyl-2-[N-(4-nitrobenzenesulfonyl)-1-phenylformamido]-N-(quinolin-3-yl)pentanamide (11t)

Yield (0.5401 g, 98.88%), brownish oil, FTIR (KBr, cm^-1^): 3388 (NH), 3065 (C-H aromatic), 2955 (C-H aliphatic), 1701, 1688 (C = O), 1631 (C = N), 1604, 1452 (C = C), 1524 (NO_2_), 1353, 1315 (2S = O), 1172, 1123 (SO_2_N), 1018 (C-N). ^1^H NMR (DMSO-d_6_, 500MHz) δ: 8.54 (d, J = 8.60 Hz, 1H, ArH), 8.43 (s, 1H, NH), 8.35 (d, J = 9.15 Hz, 2H, ArH), 8.01 (d, J = 6.90 Hz, 2H, ArH), 7.73 (d, J = 8.05 Hz, 2H, ArH), 7.57 (m, 3H, ArH), 7.46 (t, J = 7.45 Hz, 3H, ArH), 7.30 (m, 3H, ArH), 7.20 (t, J = 7.45 Hz, 1H, ArH), 7.11 (m, 3H, ArH), 3.73 (t, J = 5.70 Hz, 1H, CH-C = O), 1.57 (m, 1H, CH), 1.40 (m, 2H, CH_2_), 0.76 (m, 6H, 2CH_3_). ^13^C NMR (DMSO-d_6_, 500MHz) δ: 173.4, 167.9 (C = O), 147.3, 143.9, 142.9, 141.4, 137.9, 133.4, 131.3, 130.0, 129.8, 129.4, 129.1, 128.9, 128.7, 128.7, 126.9, 126.1, 125.8, 124.8, 124.4, 112.1 (twenty aromatic carbons), 54.7, 24.5, 23.1, 21.6, 21.4 (five aliphatic carbons). HRMS-ESI (m/z): 547.0836 (M+H), calculated, 547.0839.

#### 3-Methyl-2-[N-(4-nitrobenzenesulfonyl)-1-phenylformamido]-N-(quinolin-3-yl)pentanamide (11u)

Yield (0.5392 g, 98.72%), brownish oil, FTIR (KBr, cm^-1^): 3345 (NH), 3066 (C-H aromatic), 2959, 2876 (C-H aliphatic), 1695, 1689 (C = O), 1603 (C = N), 1584, 1451 (C = C), 1530 (NO_2_), 1350, 1314 (2S = O), 1172, 1137 (SO_2_N), 1092, 1070, 1025 (C-N). ^1^H NMR (DMSO-d_6_, 400MHz) δ: 8.33 (m, 2H, ArH), 8.01 (m, 1H, ArH), 7.91 (m, 2H, ArH), 7.73 (d, J = 8.24 Hz, 1H, ArH), 7.57 (m, 2H, ArH), 7.45 (m, 3H, ArH), 7.31 (m, 2H, ArH), 7.19 (m, 1H, ArH), 7.09 (m, 2H, ArH), 3.62 (d, J = 3.20 Hz, 1H, CH-C = O), 1.70 (m, 1H, CH), 1.32 (m, 1H, CH_a_ of CH_2_), 1.06 (m, 1H, CH_b_ of CH_2_), 0.76 (m, 6H, 2CH_3_). ^13^C NMR (DMSO-d_6_, 400MHz) δ: 172.4, 167.9 (C = O), 149.9, 147.2, 143.6, 141.1, 137.9, 133.4, 131.3, 130.0, 129.8, 129.4, 129.1, 128.7, 127.1, 126.1, 125.8, 124.8, 124.6, 112.4 (seventeen aromatic carbons), 60.9, 37.3, 24.8, 15.9, 11.4 (five aliphatic carbons). HRMS-ESI (m/z): 546.1582 (M^+^), calculated, 546.1573.

#### 3-Methyl-2-[N-(4-nitrobenzenesulfonyl)-1-phenylformamido]-N-(quinolin-3-yl)butanamide (11v)

Yield (0.5294 g, 99.39%), brownish oil, FTIR (KBr, cm^-1^): 3391 (NH), 3069 (C-H aromatic), 2961 (C-H aliphatic), 1687 (C = O), 1629 (C = N), 1604, 1584, 1452 (C = C), 1529 (NO_2_), 1351, 1316 (2S = O), 1174 (SO_2_N), 1093, 1071, 1026 (C-N). ^1^H NMR (DMSO-d_6_, 400MHz) δ: 8.34 (m, 2H, ArH), 8.01 (d, J = 8.72 Hz, 1H, ArH), 7.94 (m, 3H, ArH), 7.74 (d, J = 8.28 Hz, 1H, ArH), 7.56 (m, 2H, ArH), 7.44 (t, J = 7.80 Hz, 3H, ArH), 7.30 (m, 2H, ArH), 7.13 (m, 3H, ArH), 3.61 (d, J = 5.96 Hz, 1H, CH-C = O), 2.00 (m, 1H, CH), 0.81 (m, 6H, 2CH_3_). ^13^C NMR (DMSO-d_6_, 400MHz) δ: 172.5, 167.9 (C = O), 149.9, 147.3, 143.7, 142.9, 141.2, 137.9, 133.3, 131.3, 130.0, 129.8, 129.4, 129.1, 128.8, 128.7, 127.0, 126.1, 125.8, 124.7, 124.5, 112.4 (twenty aromatic carbons), 61.9, 30.8, 19.6, 18.2 (four aliphatic carbons). HRMS-ESI (m/z): 533.1702 (M+H), calculated, 533.1709.

#### 4-Hydroxy-1-(4-nitrobenzenesulfonyl)-N-(quinolin-3-yl)pyrrolidine-2-carboxamide (11w)

Yield (0.4421 g, 100%), appearance, white powder, mp, 80.20–80.90°C, FTIR (KBr, cm^-1^): 3428 (OH), 3332 (NH), 3054 (C-H aromatic), 2945, 2876 (C-H aliphatic), 1687 (C = O), 1641 (C = N), 1609, 1572, 1471, 1435 (C = C), 1534 (NO_2_), 1347, 1290 (2S = O), 1190, 1129 (SO_2_N), 1019 (C-N). ^1^H NMR (DMSO-d_6_, 400MHz) δ: 8.36 (d, J = 8.68 Hz, 2H, ArH), 8.02 (d, J = 8.72 Hz, 2H, ArH), 7.72 (d, J = 8.24 Hz, 1H, ArH), 7.56 (d, J = 7.80 Hz, 1H, ArH), 7.31 (m, 2H, ArH), 7.11 (m, 1H, ArH), 7.03 (s, 1H, ArH), 6.48 (s, 1H, NH), 4.17 (s, 1H, OH), 4.10 (t, J = 3.96 Hz, 1H, CH-C = O), 3.44 (m, 1H, OH), 3.19 (d, J = 5.45 Hz, 2H, CH_2_N), 2.01 (m, 1H, CH_a_ of CH_2_), 1.90 (m, 1H, CH_b_ of CH_2_). ^13^C NMR (DMSO-d_6_, 400MHz) δ: 173.5 (C = O), 150.3, 143.9, 143.6, 142.9, 129.9, 129.5, 129.0, 126.9, 126.1, 124.8, 124.5, 118.7, 112.0 (thirteen aromatic carbons), 69.0, 60.5, 57.1, 29.1 (four aliphatic carbons). HRMS-ESI (m/z): 441.0871 (M-H), calculated, 441.0872.

#### 1-(4-Nitrobenzenesulfonyl)-N-(quinolin-3-yl)pyrrolidine-2-carboxamide (11x)

Yield (0.4201 g, 98.59%), appearance, white powder, mp, 89.20–90.10°C, FTIR (KBr, cm^-1^): 3367 (NH), 3104 (C-H aromatic), 2981 (C-H aliphatic), 1691 (C = O), 1623 (C = N), 1607, 1499, 1473 (C = C), 1529 (NO_2_), 1350, 1304 (2S = O), 1199, 1163 (SO_2_N), 1106, 1011 (C-N). ^1^H NMR (DMSO-d_6_, 500MHz) δ: 8.39 (t, J = 8.60 Hz, 2H, ArH), 8.06 (d, J = 9.15 Hz, 2H, ArH), 7.72 (d, J = 8.60 Hz, 1H, ArH), 7.55 (d, J = 8.00 Hz, 1H, ArH), 7.31 (m, 2H, ArH), 7.21 (t, J = 7.45 Hz, 1H, ArH), 7.11 (m, 1H, ArH), 4.18 (dd, J = 4.00, 4.00 Hz, 1H, CH-C = O), 3.37 (m, 1H, CH_a_ of CH_2_N), 3.21 (m, 1H, CH_b_ of CH_2_N), 1.96 (m, 1H, CH of CH_2_), 1.81 (m, 2H, CH_2_), 1.63 (m, 1H, CH of CH_2_). ^13^C NMR (DMSO-d_6_, 400MHz) δ: 173.4 (C = O), 150.4, 143.9, 142.9, 141.6, 130.0, 129.2, 129.1, 128.7, 126.9, 126.0, 125.1, 124.4, 111.9 (thirteen aromatic carbons), 61.1, 48.9, 30.9, 24.7 (four aliphatic carbons). HRMS-ESI (m/z): 427.1075 (M+H), calculated, 427.1077.

### Pharmacokinetic prediction

The drug-likeness was predicted using online quantitative estimation of drug-likeness tools. The prediction was based on the calculated logP, topological polar surface area (TPSA), hydrogen bond donors (HBD), hydrogen bond acceptor (HBA), number of acid (NA), molecular weight (MW), number of rotatable bonds (NRB) and percentage absorption (ABS). The prediction tool was accessed from crdd.osdd.net/oscadd/qed/submit_virtual.php, molinspiration and druglito.

### *In vitro* antitrypanosomal activities

*In vitro* antitrypanosomal activities against *T*. *b*. *gambiense* strain was described by Otoguro *et al* [25]. In brief, *T*. *b*. *gambiense* strain was cultured in IMDM with various supplements and 10% heat-inactivated FBS at 37°C, under 5.0% CO_2_/95% air according to the method of Baltz *et al* [26]. Ninety five mL of the trypanosomes suspension (2.0–2.5104 trypanosomes/mL for strain GUTat 3.1) was seeded in a 96-well microplate, and 5.0 mL of a test compound solution (dissolved in 5.0% dimethylsulfoxide) was added followed by incubation for 72 h (long incubation-low inoculation test: LILIT). Ten mL of the fluorescent dye Alamar Blue was added to each well. After incubation for 3–6 h, the resulting solution was read at 528/20 nm excitation wavelength and 590/35 nm emission wavelength by a FLx800 fluorescent plate reader (Bio-Tek Instrument, Inc. Vermont, USA). Data were transferred into a spreadsheet program (Excel). The IC_50_ values were determined using fluorescent plate reader software (KC-4, Bio-Tek). Successive subcultures were done in 24-well tissue culture plates under the same conditions.

### Determination of LD_50_ for the active anti-inflammatory compounds

Male mice were divided into various groups and test compounds were administered in various doses intraperitoneally. Following treatments, the animals were observed for up to 4 h continuously and were then kept under observation for 72 h. All behavioral changes and deaths during the observation periods were recorded. The percentage of death at each dose level was then calculated, converted to probits and the LD_50_ (μM/kg) values were calculated [27]. To observe the health status of the mice, they were monitored 4 times a day. Humane endpoints were used when the animal shows sign of weight loss, weakness accompanied by inability to get food, complete anorexia and convulsion for 24 h. For the purpose of ameliorating the suffering of the dying mice, CO_2_ euthanasia was applied. All the dead mice were disposed in bio-safety containers in accordance with local standard protocols. The mortality rate in each group was calculated according to the formula:

Mortalityrate(%)=(thenumberofdeadmice/thenumberofmiceinthegroup)×100.

## Results and discussion

**Fig 2:**
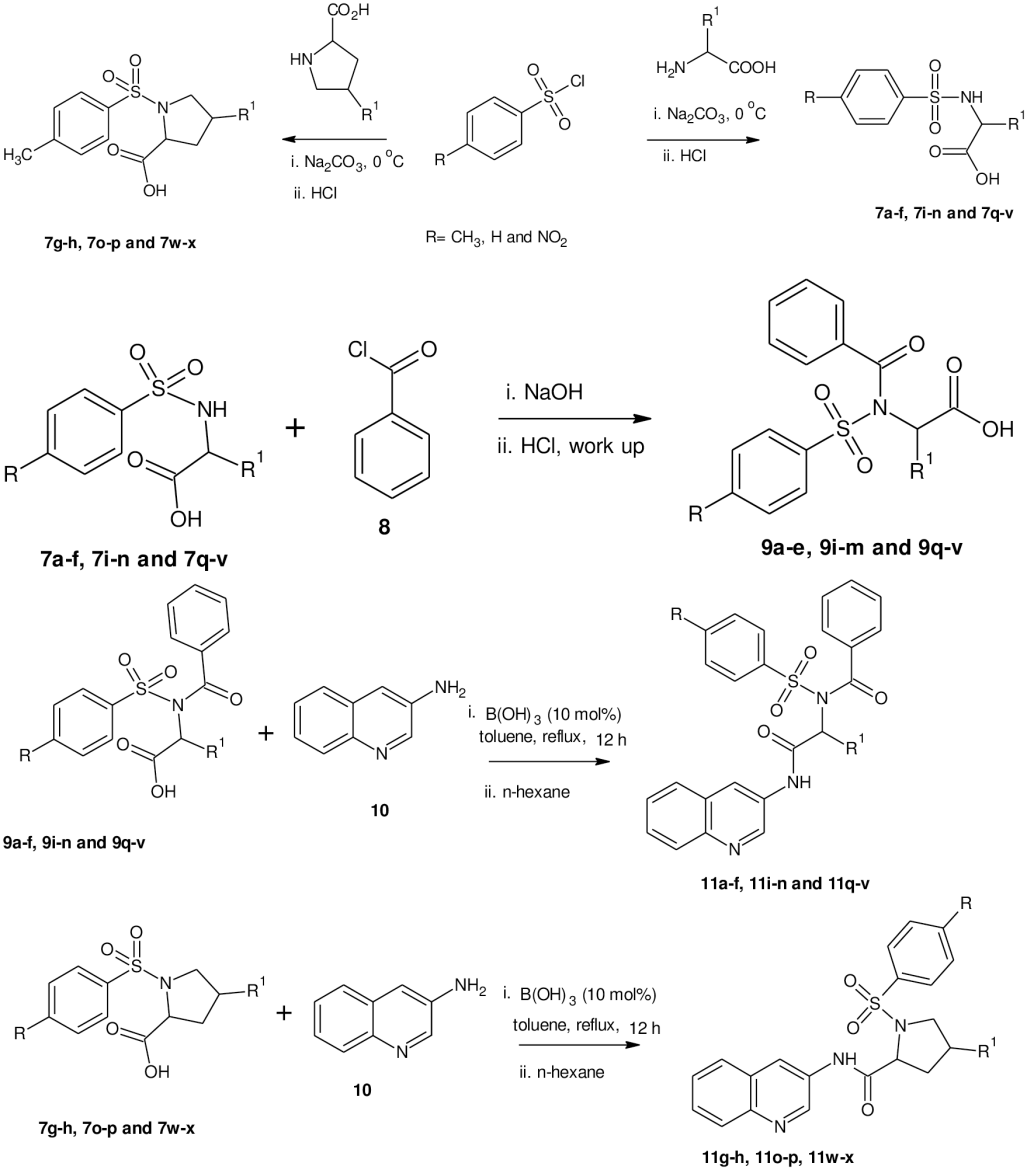
Synthesis of 3-aminoquinoline derivatives of benzenesulphonamides. A structural representation of the synthesis of benzenesulphonamides.

**Fig 3:**
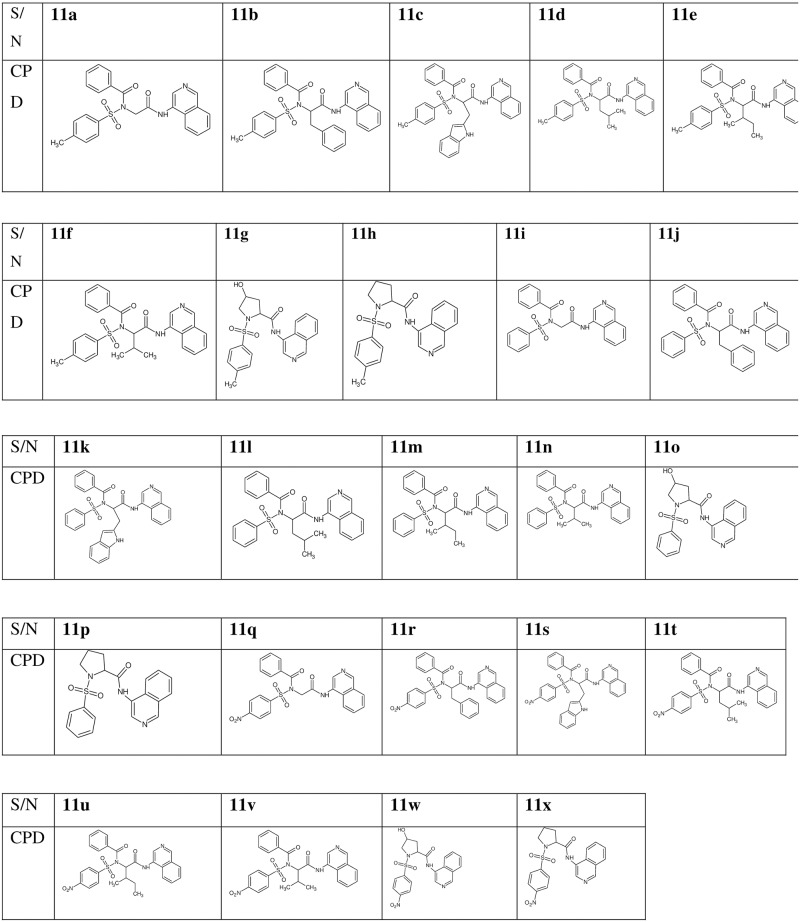
Structures of the new derivatives. A structural representation of the new derivatives.

To assess the effect of electron density, we decided to study several benzenesulphonamides bearing electron- rich and deficient substituents (Figs 2 and 3). In the first step, the substituted benzenesulphonamides (**7a-x**) were synthesized by the reaction of substituted benzenesulphonyl chlorides (**5a-c**) with amino acids (**6a-h**) in the presence of sodium carbonate as base (Fig 2). The *N*-benzoylated derivatives (**9a-f, 9i-n** and **9q-v**) were obtained by the reaction of the appropriate benzenesulphonamides (**7a-x**) with benzoyl chloride (**8**) in the presence of sodium hydroxide (Fig 2). Boric acid catalysed amidation of the carboxylic acid end of the *N*-benzoylated derivatives (**9a-f, 9i-n** and **9q-v**) and 3-aminoquinoline (**10**) afforded the corresponding carboxamide derivatives (**11a-f, 11i-n** and **11q-v**) (Fig 2). In addition to the *N*-benzoylated derivatives, the proline and 4-hydroxyproline derivatives of substituted benzenesulphonamides (**7g, h, o, p, w and x**) were also reacted with 3-aminoquinoline in the same manner to afford the carboxamide sulphonamide derivatives **11g, h, o, p, w and x**. The carboxamides were crystallized in their analytical grade using n-hexane. The synthesized compounds were characterized by various spectroscopic techniques like FTIR, ^1^H NMR, ^13^C NMR and high resolution mass spectroscopy (HRMS). The presence of a sharp bands between 3448–3415 cm^-1^, 3385–3248 cm^-1^ and 1753–1699 cm^-1^ assigned to OH of carboxylic acid, NH and C = O respectively were indicative of successful formation of compounds **7a-x**. The disappearance of the N-H band at 3385–3248 cm^-1^ in the *N*-benzoylated derivatives coupled with the appearance of an additional band at 1685–1689 cm^-1^ is diagnostic of the successful *N*-benzoylation of the substituted benzenesulphonamides. In the ^1^H NMR spectra of the *N*-benzoylated derivatives, the disappearance of the NH peak and the appearance of additional aromatic peaks having five protons were indicative of successful coupling. In the ^13^C NMR, the appearance of four additional aromatic carbons and a peak at 167 ppm were diagnostic of successful *N*-benzoylation.

The IR of all the new carboxamides showed a sharp band between 3452–3359 cm^-1^ and 1632–1622 cm^-1^ corresponding to the NH and C = N band respectively. In the ^1^H NMR, the singlet appearing between 8.46–8.40 ppm in the derivatives corresponds to the one aromatic proton of 3-aminoquinoline group; the peak at 6.54–6.50 ppm corresponds to the singlet peak of the NH group in the carboxamide derivatives. In the ^13^C NMR spectra, the appearance of additional nine aromatic peaks corresponds to the nine carbons of the 3-aminoquinoline. These spectral characterizations, in addition to the HRMS-ESI spectra which showed the molecular ion peaks of the compounds, were supportive of the successful formation of the final products. The aromatic and aliphatic protons and carbons appeared at the predicted range using BioDraw.

### *In vitro* antitrypanosomal activities and structure-activity relationship

**Fig 4:**
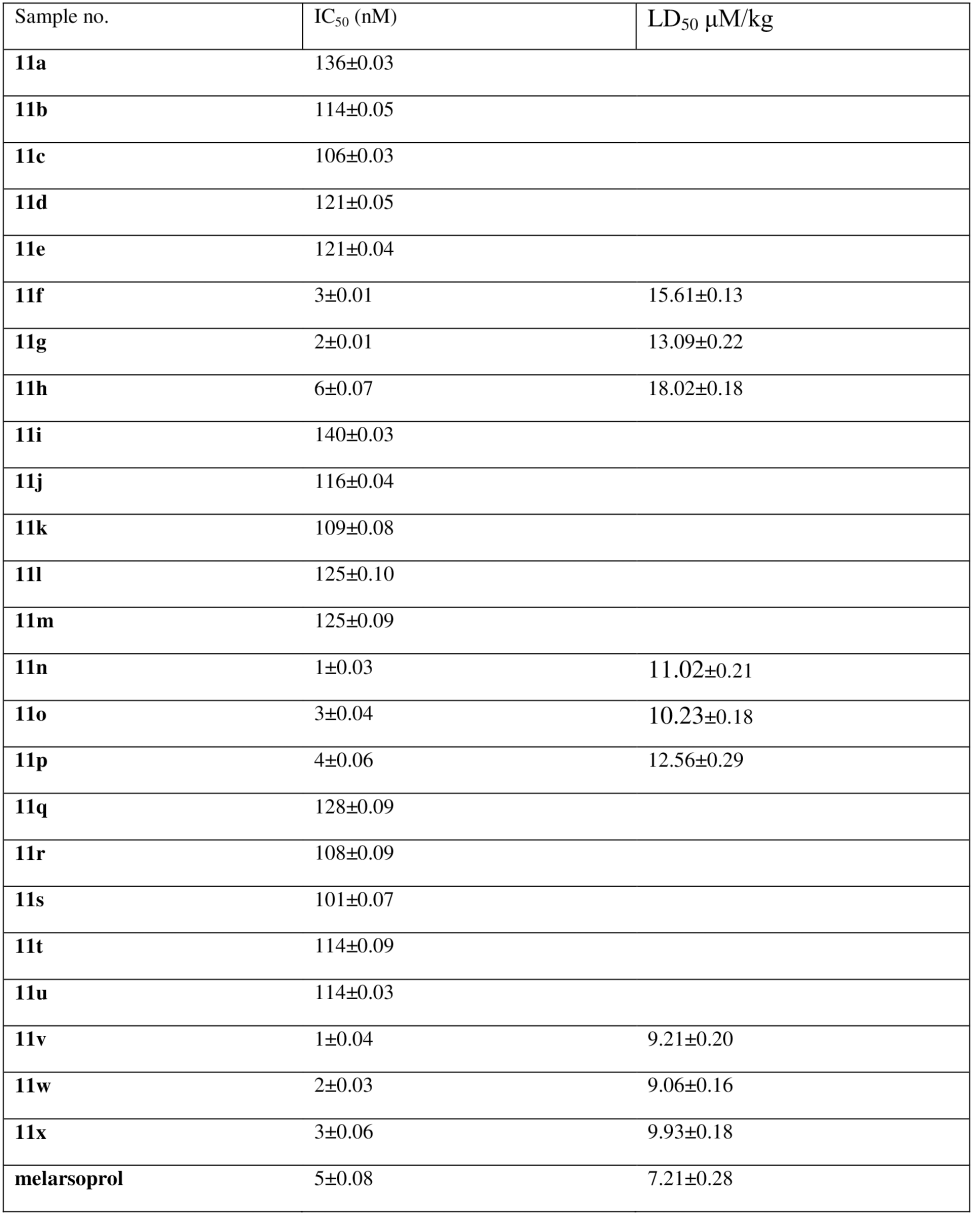
*In vitro* antitrypanosomal activities. A table of value representing the *in vitro* antitrypanosomal activities.

The results of the *in vitro* antitrypanosomal activities (Fig 4), revealed that compounds **11f, 11g, 11h, 11n, 11o, 11p, 11v, 11w** and **11x** had IC_50_ values of 3, 2, 5, 1, 3, 4, 1, 2, 3 nM respectively which in exception of compounds **11h** and **11p** were 2-fold, 3-fold, 5-fold, 2-fold, 5-fold, 3-fold and 2-fold more active than melarsoprol (IC_50_ 5 nM). These derivatives with good antitrypanosomal activity against *T*. *b*. *gambiense* could serve either as a replacement to melarsoprol and pentamidine where there is record of toxicity or as a new entrant to HAT chemotherapy. The L-valine, L-hydroxyproline and L-proline derivatives were the most active compounds in each of the series. The 4-nitrobenzenesulphonamide derivatives were found to be more active than the unsubstituted benzenesulphonamide and 4-methylbenzenesulphonamide derivatives. The *p*-nitrobenzenesulphonamides were the most active derivatives followed by the *p*-toluenesulphonamides and then the benzenesulphonamides. These findings underscores the importance of a substituent at the para position of the benzene ring in the antitrypanosomal activity. In general, the effect of the substituents on the benzene ring followed the trend NO_2_>CH_3_>H. The effect of the amino acid residue showed that the L-valine derivatives (**11f, 11n** and **11v**) had better activity than the L-leucine (**11d, 11l** and **11t**), L-isoleucine (**11e, 11m** and **11u**) and glycine (**11a, 11i** and **11q**) derivatives. This shows that the chain length of a substituent at the β-carbon contributes significantly to biological activity. The effect of the presence of a substituent at the β-carbon was shown by the biological activity of the L-leucine, L-isoleucine and L-valine derivatives which were better than the glycine derivatives that lacked substitution at the β-carbon. Among the aromatic amino acids, the L-tryptophan (**11c, 11k** and **11s**) were more active than the L-phenylalanine (**11b, 11j** and **11r**), this may not be unconnected with the presence of nitrogen on the ring. The L-4-hydroxyproline (**11g, 11o** and **11w**) were more active than their corresponding L-prolines (**11h, 11p** and **11x**) which highlights the importance of the hydroxyl group on biological activity of the molecules.

The LD_50_ values showed that the most promising hits in exception of the *p*-nitrobenzenesulphonamide derivatives (**11v-x**) had better toxicity profile than melarsoprol and as such may likely not have the toxicity problems associated with melarsoprol and other antitrypanosomal agents currently in use. Compound **11h** was the safest hit with LD_50_ of 18.02 μM/kg. The *p*-toluenesulphonamide derivatives (**11f-h**) were the safest derivatives followed by the benzenesulphonamide derivatives (**11n-p**).

### *In silico* calculation of the pharmacokinetics properties (ADME-T) and drug-likeness

Drug-likeness describes an integrated equilibrium between multiple molecular properties and structural features that define whether a particular compound is comparable to known drugs. Lipinski’s rule of 5 (ro5) is among the most common principle applied to evaluate drug-likeness properties of a compound [28]. Verber’s criteria have also been applied extensively to evaluate oral bioavailability of compounds [29]. These properties measure hydrophilicity, electronic distribution, hydrogen bonding capability, molecule size and flexibility that would affect molecules behaviours in a living system. The behavior predicted includes bioavailability, transport properties, affinity to proteins reactivity and toxicity [30].

A computational studies of all the carboxamides **11a-x** using DrugLiTo and MedChem Designer 3.0 software was carried out to determine the Lipinski’s molecular properties, the blood-brain barrier likeness, and the number of rotatable bonds (NRB), together with the topological polar surface area (TPSA; a sum of polar atoms surfaces, a descriptor for drug absorption, penetrability and bioavailability) and the percentage of absorption (ABS %) calculated as (ABS % = 109–0.345×TPSA) [31].

**Fig 5:**
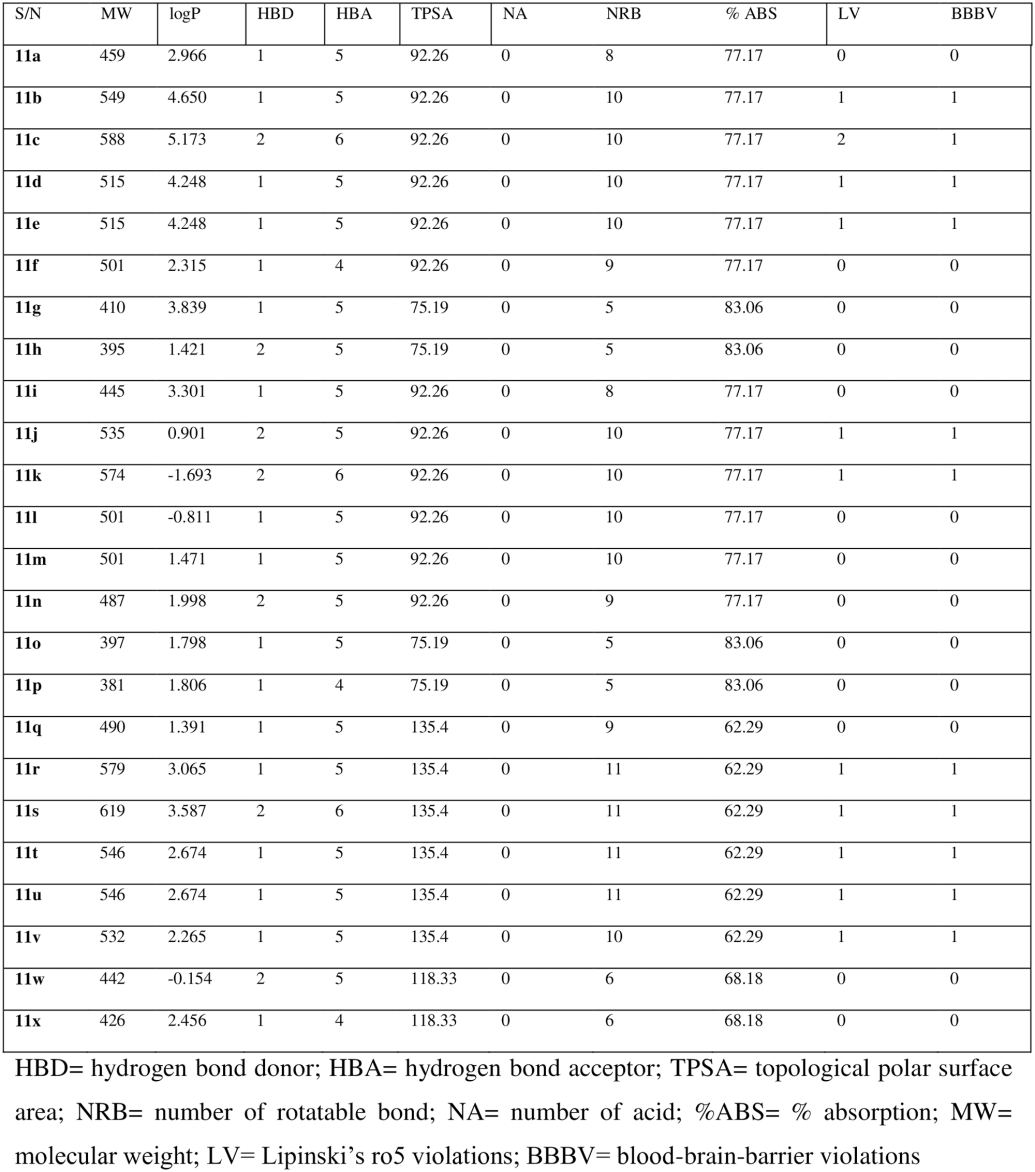
Pharmacokinetic properties *in vitro* antitrypanosomal activities. A table of value repreenting the *in vitro* antitrypanosomal activities.

The results of the pharmacokinetics prediction (Fig 5) revealed that all the tested compounds in exception of compound **11c** complied with ro5 where LogP values ranged between -0.154 and 4.650 (≤ 5), MW range 381–619 (≤ 500 but one violation is permissible for ro5), HBA range 4–6 (≤10), HBD range 1–2 (≤5), suggesting that these compounds would not be expected to cause oral bioavailability problems. Compound **11c** needs derivatization to improve on its oral bioavailability profile. In exception of compounds **11r-11u** (NRB = 11), all the compounds showed NRB values range 5–10 (≤10), indicating acceptable molecular flexibility with consequent expected good permeability and oral bioavailability. Additionally, all the compounds evaluated showed TPSA range 75.19–135.40 Å^2^ (≤140 Å^2^), showing good permeability and transport of the compounds in the cellular plasma membrane. Furthermore, all the tested compounds exhibited a considerable %ABS range 62.29–83.06% which is a designation of good bioavailability upon oral administration. Finally, since sleeping sickness occurs when single-cell trypanosome parasites spread from to brain over the blood-brain barrier, the BBB likeness of the compounds were evaluated. All the evaluated compounds showed NA values 0 (0), HBA range 4–6 (˂10) and MW range 381–619 (˂500), indicating that compounds **11b-11e, 11j-11k** and **11r-11v** cannot cross the blood-brain barrier and as such would not succeed in the treatment of second stage of human African trypanosomiasis. It is important to note that the more active derivatives in the *in vitro* antitrypanosomal activities study (**11f-h, 11n-p** and **11v-x**) had fascinating pharmacokinetics properties.

## Conclusion

Despite the fact that over 100,000 death annually is attributed to trypanosome, there is currently no new drug for the treatment of trypanosome. Although eflornithine was introduced in the 90’s, it was found to be ineffective against *T*. *b*. *rhodesiense*. The development of resistance species to existing antitrypanosomal agent is quite worrisome. We have presented an efficient, eco-friendly, highly yielding and versatile direct amidation procedure for unactivated carboxylic acids bearing benzenesulphonamides. Beside the synthetic procedure reported, the compounds were found to possess fascinating antitrypanosomal activities against *T*. *b*. *gambiense*. Some of these derivatives reported herein was found to be more active than melarsoprol. The novel compounds showed satisfactory predicted physico-chemical properties including oral bioavaiability, permeability and transport properties.

## Supporting information

S1 File ^1^H and ^13^C spectra of the new derivatives. (DOC)
