# Supplementary material for: Synthesis, characterization and in vitro antitrypanosomal activities of new carboxamides bearing quinoline moiety
Source: PLoS One. 2018 Jan 11;13(1):e0191234. doi: 10.1371/journal.pone.0191234 (PMC5764481; doi:10.1371/journal.pone.0191234)
Supplement: S1 File — (DOC) [file pone.0191234.s001.doc]

**^1^H and ^13^C NMR spectra of the new derivatives**

**11a**

**11b**

**11c**

**11d**

**11e**

**11f**

**11g**

**11h**

**11i**

**11j**

**11k**

**11l**

**11m**

**11n**

**11p**

**11p**

**11q**

**11r**

**11s**

**11t**

**11u**

**11v**

**11w**

**11x**
